# Structural and Functional Insights into the HIV-1 Maturation Inhibitor Binding Pocket

**DOI:** 10.1371/journal.ppat.1002997

**Published:** 2012-11-08

**Authors:** Kayoko Waki, Stewart R. Durell, Ferri Soheilian, Kunio Nagashima, Scott L. Butler, Eric O. Freed

**Affiliations:** 1 Virus-Cell Interaction Section, HIV Drug Resistance Program, Frederick National Laboratory for Cancer Research, National Cancer Institute, Frederick, Maryland, United States of America; 2 Laboratory of Cell Biology, Center for Cancer Research, National Cancer Institute, National Institutes of Health, Bethesda, Maryland, United States of America; 3 Electron Microscope Laboratory, Advanced Technology Program, SAIC-Frederick, Frederick National Laboratory for Cancer Research, National Cancer Institute, Frederick, Maryland, United States of America; 4 Antiviral Biology, Pfizer Global Research & Development, Sandwich Laboratories, Sandwich, Kent, United Kingdom; Universitätklinikum Heidelberg, Germany

## Abstract

Processing of the Gag precursor protein by the viral protease during particle release triggers virion maturation, an essential step in the virus replication cycle. The first-in-class HIV-1 maturation inhibitor dimethylsuccinyl betulinic acid [PA-457 or bevirimat (BVM)] blocks HIV-1 maturation by inhibiting the cleavage of the capsid-spacer peptide 1 (CA-SP1) intermediate to mature CA. A structurally distinct molecule, PF-46396, was recently reported to have a similar mode of action to that of BVM. Because of the structural dissimilarity between BVM and PF-46396, we hypothesized that the two compounds might interact differentially with the putative maturation inhibitor-binding pocket in Gag. To test this hypothesis, PF-46396 resistance was selected for *in vitro*. Resistance mutations were identified in three regions of Gag: around the CA-SP1 cleavage site where BVM resistance maps, at CA amino acid 201, and in the CA major homology region (MHR). The MHR mutants are profoundly PF-46396-dependent in Gag assembly and release and virus replication. The severe defect exhibited by the inhibitor-dependent MHR mutants in the absence of the compound is also corrected by a second-site compensatory change far downstream in SP1, suggesting structural and functional cross-talk between the HIV-1 CA MHR and SP1. When PF-46396 and BVM were both present in infected cells they exhibited mutually antagonistic behavior. Together, these results identify Gag residues that line the maturation inhibitor-binding pocket and suggest that BVM and PF-46396 interact differentially with this putative pocket. These findings provide novel insights into the structure-function relationship between the CA MHR and SP1, two domains of Gag that are critical to both assembly and maturation. The highly conserved nature of the MHR across all orthoretroviridae suggests that these findings will be broadly relevant to retroviral assembly. Finally, the results presented here provide a framework for increased structural understanding of HIV-1 maturation inhibitor activity.

## Author Summary

Maturation of HIV-1 particles, which occurs as they bud off from the infected cell, is triggered by the step-wise cleavage of the major viral structural polyprotein, Pr55Gag, to individual, mature Gag proteins. The viral protease is the enzyme responsible for Gag polyprotein cleavage. Maturation inhibitors prevent the viral protease from processing Gag at one particular cleavage site, but how they accomplish this is not understood. In this study, the ability of HIV-1 to become resistant to the two structurally distinct maturation inhibitors that have thus far been reported was examined. We found that one of these compounds, PF-46396, gives rise to resistance mutations that map to three domains in Gag, including a region known as the major homology region (MHR). The MHR is highly conserved among retroviruses and is known to be very important for virus assembly and maturation. These MHR mutants were observed to replicate much better in the presence of PF-46396 than in its absence; i.e., these mutants are compound-dependent. We were also able to select for second-site mutations in Gag that reversed the replication defects imposed by the MHR mutations. These results define residues in Gag that comprise the maturation inhibitor-binding pocket and also identify regions of Gag that structurally and functionally interact with the MHR.

## Introduction

Over 20 antiretroviral inhibitors have been approved for clinical use in HIV-1-infected patients. These drugs fall into several classes, mostly targeting the viral enzymes reverse transcriptase (RT), protease (PR), and integrase (IN). A fusion inhibitor specific for the viral transmembrane envelope (Env) glycoprotein gp41, and an entry inhibitor directed against the viral coreceptor CCR5 are also available. These antiretrovirals, administered in combinations referred to as highly active antiretroviral therapy (HAART), are quite effective and have resulted in striking declines in AIDS-related mortality in treated patients [1], [2], [3], [4]. However, resistance to these compounds, as well as a variety of drug tolerability and related compliance issues, have reduced their benefit in many patients. Given that resistance is likely to become an increasingly large problem over time, discovering new inhibitors that target distinct steps in the viral replication cycle remains a high priority [5]. In addition to the potential therapeutic benefit of such new antiretrovirals, drug discovery efforts are likely to provide novel and important insights into the molecular biology of HIV-1 replication.

A promising but still underdeveloped class of anti-HIV-1 compounds are the maturation inhibitors [6]. Maturation is an essential step in the virus replication cycle that is triggered during or shortly after virus release from the infected cell by the PR-mediated processing of the Gag and GagPol polyprotein precursors. The Gag precursor, Pr55^Gag^, is cleaved into the following mature Gag proteins and intervening spacer peptides (SPs): matrix (MA), capsid (CA), SP1, nucleocapsid (NC), SP2, and p6 [7], [8]. The GagPol precursor, Pr160^GagPol^, gives rise to the mature viral enzymes PR, RT, and IN. Because of the unique sequence and context of each of the processing sites in Pr55^Gag^ and Pr160^GagPol^, PR cleaves each site at a different rate, leading to a step-wise proteolytic cascade [9], [10], [11]. Maturation involves a major conformational rearrangement of viral proteins within the virion. Most notably, CA reorganizes to form a conical shell that surrounds the viral RNA genome. This conical shell, which exhibits fullerene-like geometry, is constructed from a hexagonal lattice of CA monomers closed off at either end by the incorporation of a specific number of pentamers [8], [12], [13]. CA is composed of two structural domains: the N-terminal and the C-terminal domain (NTD and CTD, respectively), which are connected by a short, flexible linker. Located just downstream of the interdomain linker at the N-terminus of the CA-CTD is a ∼20 amino acid sequence known as the major homology region (MHR) that is highly conserved across orthoretroviral capsid proteins [14], [15], [16]. The MHR adopts a strand/turn/helix fold that likely functions in forming the immature Gag lattice during virus assembly [17]. Previous mutational analyses have demonstrated that disruption of the MHR impairs assembly, maturation, and infectivity of a number of retroviruses including HIV-1, Mason-Pfizer monkey virus, Rous sarcoma virus, and bovine leukemia virus [14], [18], [19], [20], [21], [22], [23], [24], [25], [26]. Early X-ray crystallography data indicate that the HIV-1 MHR forms a network of hydrogen bonds involving CA residues R154, Q155, G156, E159, and R167 [17]. SP1, located downstream of CA, has been proposed to adopt a helical conformation [27]. Based on cryo-electron tomography reconstructions, SP1 is suggested to form six-helix bundles that stabilize each CA hexamer [28]. Some mutations in SP1 disrupt virus assembly, consistent with this region of Gag playing a role in promoting Gag-Gag interactions during virus particle production [29], [30], [31], [32], [33], [34].

We and others have characterized the first-in-class maturation inhibitor [3-O-(3′, 3′-dimethylsuccinyl) betulinic acid [known variously as PA-457, bevirimat (BVM), or DSB] [35], [36], [37], [38], [39]. Treatment of virus-producing cells with BVM leads to the accumulation of CA-SP1, indicating that the compound blocks cleavage of this processing intermediate to mature CA. It is noteworthy that BVM disrupts but does not completely block CA-SP1 processing; some mature CA is generated even at high concentrations of the compound. Propagation of HIV-1 in the presence of BVM in culture gives rise to resistance mutations in the immediate vicinity of the CA-SP1 cleavage site [38], [39], [40], [41]. It is currently hypothesized that BVM binds a pocket located near the CA-SP1 cleavage site, a region for which little high-resolution structural information is available. Direct binding of radiolabeled BVM to immature but not mature HIV-1 particles has been measured [42], suggesting that the putative binding pocket is eliminated by PR cleavage of the Gag precursor. Some BVM resistance mutations reduced compound binding to immature particles, indicating that at least in some cases resistance is associated with disruption of the putative compound binding pocket [42], [43]. Gag assembly is required for BVM binding; the compound is able to block PR-mediated CA-SP1 processing in the context of *in vitro*-assembled Gag but does not disrupt cleavage of non-assembled Gag [38], [44]. A recent study using photoactivatable BVM derivatives observed direct cross-linking of the compound to residues in the vicinity of the CA-SP1 cleavage site, supporting the hypothesis that BVM interacts closely with this region of Gag [45]. Interestingly, some BVM cross-linking was also detected upstream in the MHR [45]. However, no resistance mutations arose in the MHR during extensive selection experiments [40].

BVM performed well in clinical trials, with significant reductions in viral loads in approximately half of treated patients [46], [47]. Unfortunately, naturally occurring polymorphisms within SP1 downstream of the CA-SP1 cleavage site (in the so-called “QVT motif” comprising SP1 residues 6–8) were associated with lack of response in other patients [48], [49], [50]. *In vitro* assays confirmed that SP1 polymorphisms, particularly SP1-V7A, reduced sensitivity of HIV-1 to BVM [51], [52]. This lack of response in a significant percentage of treated patients led to discontinuation of efforts to develop BVM for clinical use.

**Figure 1:**
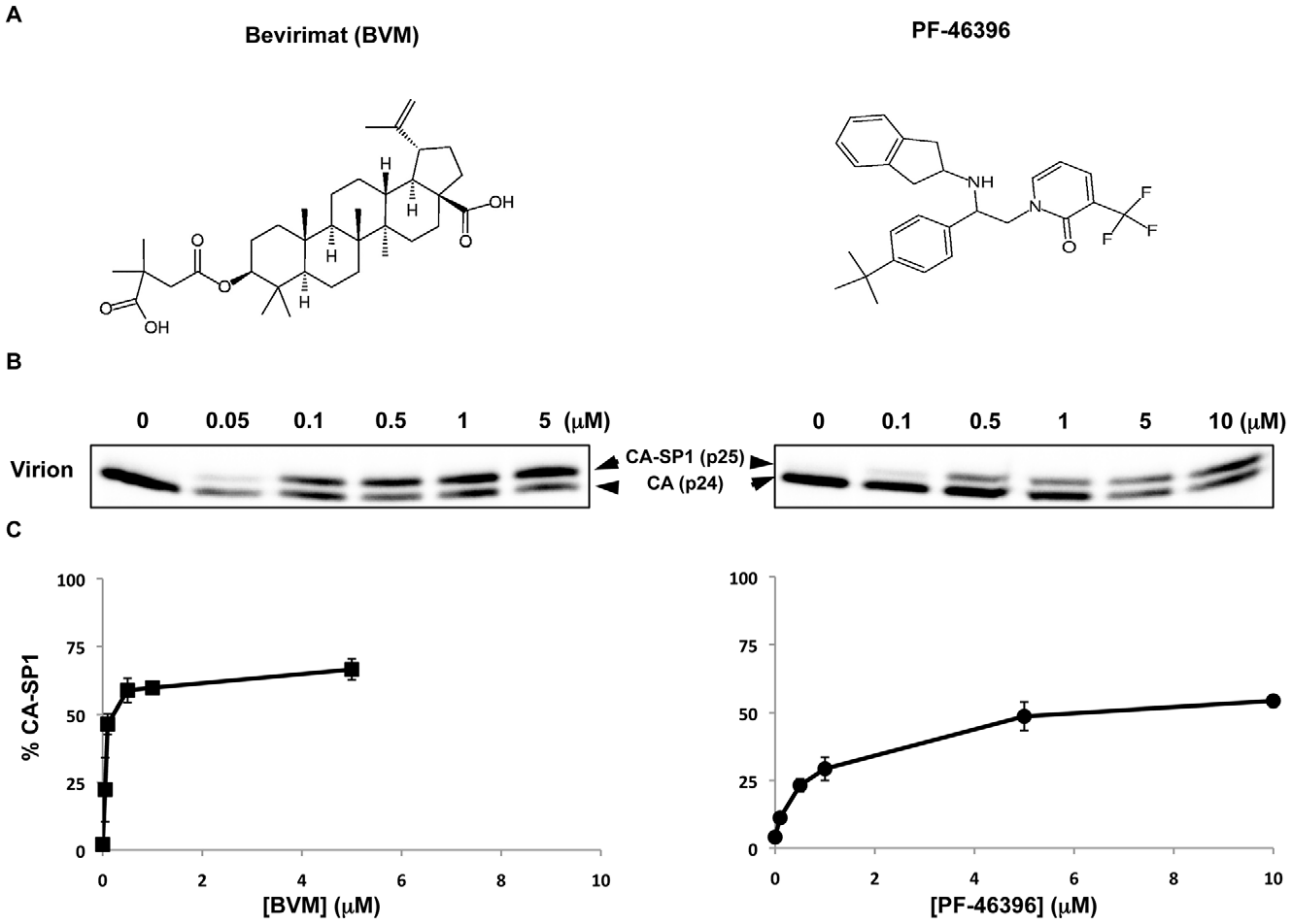
PF-46396 blocks CA-SP1 processing. [A] Chemical structures of BVM and PF-46396. [B] Radioimmunoprecipitation analysis of virion-associated CA and CA-SP1 in the presence of increasing concentrations of BVM or PF-46396. [C] Quantification of the % CA-SP1 relative to total CA+ CA-SP1 in virion fraction at the indicated concentrations of BVM or PF-46396. Error bars indicate SD; N>3.

In 2009, Blair and colleagues reported that a second compound, {1-[2-(4-tert-butylphenyl)-2-(2,3-dihydro-1H-inden-2-ylamino)ethyl]-3-(trifluoromethyl)pyridin-2(1H)-one} (PF-46396), blocked CA-SP1 processing [53]. A single PF-46396-resistance mutation was identified during selection experiments: CA-I201V, located 30 amino acids upstream of the CA-SP1 cleavage site. Intriguingly, BVM and PF-46396 are structurally distinct (Fig. 1A), indicating that compounds from different chemical classes can interfere with CA-SP1 processing. A key question is therefore whether BVM and PF-46396 bind distinct pockets on the assembled Gag multimer or whether they occupy the same binding site.

In this study, we sought a more in-depth understanding of the structure-function correlates of maturation inhibitor activity. We first examined whether combined treatment with BVM and PF-46396 would result in a more complete disruption of CA-SP1 processing than observed with either compound alone. Whether the activity of PF-46396 is reduced by polymorphisms in the SP1 QVT motif, as observed for BVM, was also investigated. Currently, no high-resolution structures of the CA-SP1 junction are available, and the precise binding site(s) of maturation inhibitors are not known, hence extensive selection experiments were performed to capture the full range of mutations that confer resistance to PF-46396. In addition to resistance mutations near the CA-SP1 cleavage site that HIV-1 acquires during propagation in BVM, a cluster of mutations in the CA MHR far upstream of the CA-SP1 junction was identified. These MHR mutants exhibited a striking degree of compound dependence and could revert by acquiring second-site changes downstream in CA and in SP1. The results of this study offer new insights into maturation inhibitor activity and the determinants of maturation inhibitor binding. The findings reported here also provide novel information about the CA-CTD and SP1 in HIV-1 assembly and maturation.

## Results

### Comparison of the effect of BVM and PF-46396 on CA-SP1 processing

In 2009, PF-46396 was described as a maturation inhibitor, which, like BVM, disrupts the cleavage of the CA-SP1 processing intermediate to mature CA [53]. To confirm this finding, and to compare the activity of the two compounds, 293T cells were transfected with the HIV-1 molecular clone pNL4-3, and metabolically radiolabeled the transfected cells in the presence of compounds. The levels of CA-SP1 in virion fractions were determined by quantitative radioimmunoprecipitation (radio-IP). As indicated in Fig. 1B and C, both BVM and PF-46396 treatment of virus-producing cells led to a dose-dependent accumulation of CA-SP1 in virions. Similar accumulation of CA-SP1 was also detected in cell-associated fractions (data not shown). The shape of the dose-response curve (Fig. 1C) indicated that PF-46396 is less potent than BVM. For both compounds, CA-SP1 accumulation reached a plateau; this occurred at around 0.5 µM for BVM and 5 µM for PF-46396. These results are consistent with prior calculation of the EC_50_ of BVM and PF-46396 [38], [53].

We previously demonstrated that BVM treatment led to the production of virus particles with an aberrant morphology typified by the presence of an electron-dense crescent of Gag located just inside the viral envelope and an aggregate of electron density located in an acentric position in the virion [38], [40]. This electron-dense crescent represents a remnant of the immature Gag lattice [54]. To investigate the effect of PF-46396 treatment on virion morphogenesis, we performed thin section transmission electron microscopy (EM) on pNL4-3-transfected cells treated with the compound. Untreated and BVM-treated samples were included as controls. Virions from untreated cells showed typical electron-dense conical cores (Fig. S1, panel a), whereas virus from BVM-treated cells displayed the previously described [38], [40] morphology (Fig. S1, panel b). Some virions produced from PF-46396-treated cells showed a morphology similar to that of particles from BVM-treated cells (e.g., Fig. S1, panel c, upper right). Interestingly, other particles produced from PF-46396 treated cells displayed a hybrid-type morphology, with a conical core and the electron-dense crescent (blue arrow, Fig. S1, panel c, lower left). Thus, particles from PF-46396-treated cells show greater morphological heterogeneity than those produced from BVM-treated cells (Fig. S1, panels c–g).

### SP1 polymorphisms have a minimal effect on susceptibility to PF-46396

**Figure 2:**
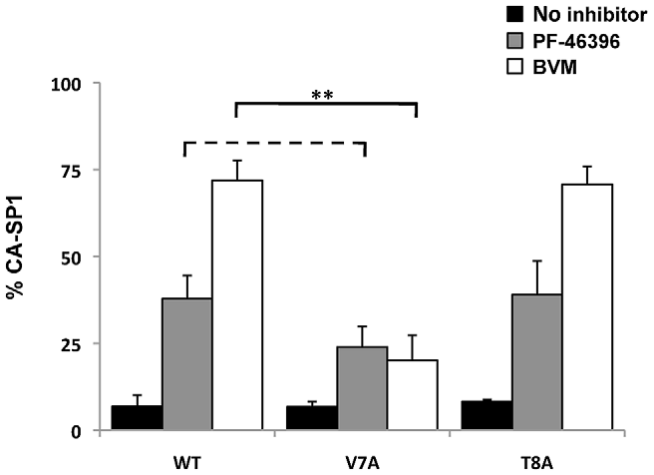
The SP1-V7A polymorphism has a lesser effect on sensitivity to PF-46396 than to BVM. 293T cells were transfected with WT pNL4-3 or pNL4-3 derivatives bearing SP1-V7A or SP1-T8A polymorphisms and treated with 1 µM BVM or PF-46396. CA-SP1 processing efficiency was examined in virions by radioimmunoprecipitation analysis. P values: **, p<0.01; dashed line, no significant difference. Error bars indicate SD; N = 3.

As mentioned in the Introduction, we and others previously reported that polymorphisms in SP1, specifically in the QVT sequence (SP1 residues 6–8), markedly reduce the sensitivity of HIV-1 to BVM. In particular, the SP1-V7A change almost completely abrogated the ability of BVM to block CA-SP1 processing. To test whether the SP1-V7A mutation similarly diminished the potency of PF-46396, we compared the effect of BVM and PF-46396 on CA-SP1 processing for SP1-V7A (Fig. 2). As controls WT and SP1-T8A, a mutant that exhibited full susceptibility to BVM [5] were included. At compound concentrations of 1 µM, WT virus particles from BVM-treated cells showed a ∼70% accumulation of CA-SP1, whereas WT particles from PF-46396-treated cells contained ∼35% CA-SP1. Consistent with our earlier report [5], under these conditions, markedly less (∼20%) CA-SP1 was detected in V7A particles produced in the presence of BVM. In contrast, the V7A mutation had a much smaller, and statistically insignificant, effect on the ability of PF-46396 to block CA-SP1 processing. We also observed that PF-46396 significantly delayed the replication kinetics of V7A (data not shown), consistent with the biochemical analysis demonstrating that V7A CA-SP1 processing is sensitive to PF-46396 (Fig. 2). As we reported previously [5], the SP1-T8A mutation did not affect sensitivity to BVM; this mutation likewise did not alter the susceptibility to PF-46396. Together, these results demonstrate that while PF-46396 is somewhat less potent than BVM, its activity is less affected by SP1 polymorphisms than that of BVM.

### PF-46396 antagonizes the activity of BVM when cells are treated simultaneously with both compounds

**Figure 3:**
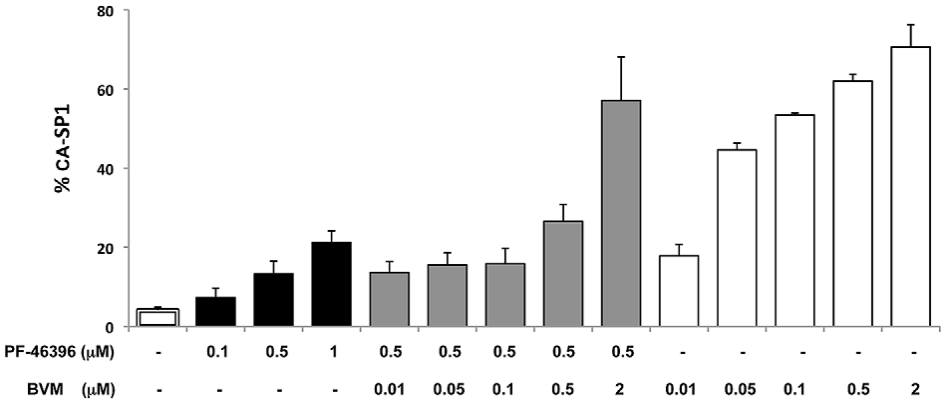
PF-46396 interferes with BVM activity at low BVM concentration. 293T cells were transfected with WT pNL4-3 and treated with either PF-46396 alone, BVM alone, or PF-46396+BVM at the indicated concentrations. Levels of CA-SP1 in virion fractions were evaluated by radioimmunoprecipitation analysis. Error bars indicate SD; N = 3.

A notable feature of both BVM and PF-46396 is that their activity plateaus at ∼70% CA-SP1 accumulation for BVM and ∼60% for PF-46396 (e.g., Fig. 1B and C). The structural dissimilarity between BVM and PF-46396 (Fig. 1A) raises the possibility that these two maturation inhibitors might occupy different pockets on the assembled Gag complex. If this were the case, the two compounds could potentially act in an additive or synergistic fashion, providing greater inhibition of CA-SP1 processing than achievable with either compound alone. To test this possibility, virus-producing cells were treated with 0.1–1.0 µM PF-46396, 0.01–2.0 µM BVM, or simultaneously with a constant amount (0.5 µM) of PF-46396 and an increasing concentration (0.01–2.0 µM) of BVM. Consistent with the data in Fig. 1, both PF-46396 and BVM inhibited CA-SP1 in a dose-dependent manner when either was added alone (Fig. 3). In contrast, the presence of 0.5 µM PF-46396 prevented escalating concentrations of BVM from increasing CA-SP1 accumulation. For example, at 0.05 µM BVM alone, an accumulation of ∼45% CA-SP1 was observed, whereas at the same concentration of BVM and 0.5 µM PF-46396 only ∼15% CA-SP1 accumulated. Similarly, treatment with 0.1 µM BVM resulted in ∼50% CA-SP1 accumulation, whereas this concentration of BVM in the presence of 0.5 µM PF-46396 again led to ∼16% CA-SP1 accumulation. The antagonistic behavior of PF-46396 towards BVM activity could be superseded at high concentrations of BVM, such that at 2 µM BVM, even in the presence of 0.5 µM PF-46396, ∼60% CA-SP1 accumulation was observed (Fig. 3).

### PF-46396 resistance maps to three domains in Gag

**Figure 4:**
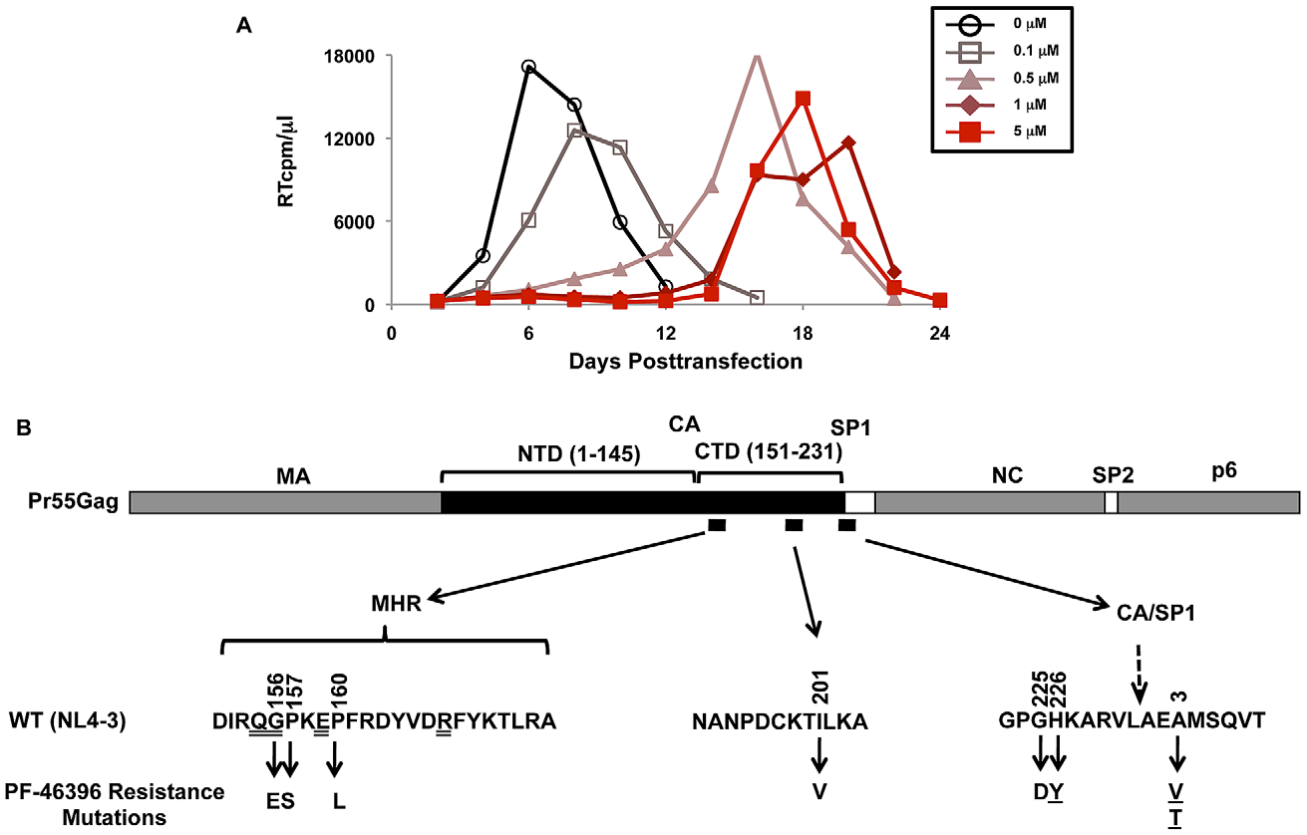
PF-46396 resistance mutations map to three regions within the CA-CTD and SP1. [A] Example of a selection experiment for PF-46396 resistance. The Jurkat T-cell line was transfected with pNL4-3 and propagated in the presence of 0–5 µM PF-46396. Virus replication was monitored by RT activity, shown in counts per minute (cpm) per µl of culture medium. [B] Location of PF-46396 resistance mutations in Gag. A linear diagram of Pr55^Gag^ is provided, with CA N-terminal and C-terminal domains (NTD and CTD, respectively) indicated. Small black boxes denote the location in Gag of the resistance mutations, which are shown below using CA and SP1 amino acid numbers. Single-underlined mutations represent those that we previously obtained during BVM selections; highly conserved residues in the MHR are denoted by double underlines.

To examine the effect of PF-46396 on the establishment of a spreading HIV-1 infection, the Jurkat T-cell line was transfected with pNL4-3 in the absence of the compound or at concentrations ranging from 0.1 to 10 µM. In the experiment shown in Fig. 4A, and in a number of repeat experiments, virus replication, as measured by reverse transcriptase (RT) activity in the culture medium, peaked approximately six days posttransfection in the absence of compound. However, at PF-46396 concentrations of 0.5 µM or higher, virus replication was significantly delayed, with peak RT levels occurring at around three weeks posttransfection in 5 (Fig. 4A) and 10 µM (data not shown) compound.

Based on the reasoning that identification of PF-46396 resistance mutations would provide key insights into the residues that line the PF-46396 binding pocket, we determined whether the virus replicating with delayed kinetics had acquired PF-46396 resistance. Viral genomic DNA was purified at the peak of RT activity, amplified Gag and PR coding regions by PCR, and sequenced the PCR products. Five mutations were found: three (CA-G225D, CA-H226Y, and SP1-A3V) located near the CA-SP1 cleavage site, and two (CA-G156E and CA-P157S) within the CA MHR (Fig. 4B). The CA-H226Y and SP1-A3V mutations were previously identified in our selections for BVM resistance [40]. Similar replication experiments were performed with pNL4-3 clones bearing common polymorphisms in residues 6–8 of SP1 [51]. Several of the mutations that emerged during propagation of WT NL4-3 were again observed in these selection experiments (Table 1). In addition, several other changes were found: CA-P160L, CA-I201V, and SP1-A3T. CA-I201V was previously identified in selection experiments for PF-46396 resistance [53], and we previously selected for SP1-A3T during NL4-3 propagation in the presence of BVM [40]. Together, these results demonstrate that propagation in PF-46396 led to the selection of changes not only in the vicinity of the CA-SP1 cleavage site where BVM resistance maps, but also upstream in CA at residue 201 and in the MHR. The appearance of mutations in the MHR is particularly intriguing, given the essential nature of this domain in HIV-1 assembly and maturation (see Introduction).

**Table 1:** Summary of PF-46396-resistance mutations selected.

Viruses Used in Selections #	PF-46396-resistance Mutations
WT	CA-G156E
	CA-P157S (2)*
	CA-H226Y
	CA-G225D (2)
	SP1-A3V
SP1-Q6A	CA-P160L
	CA-I201V
SP1-V7M	SP1-A3T
	CA-G156E
	CA-P157S
SP1-V7A	CA-H226Y
SP1-T8A	CA-G225D
	SP1-A3T
SP1-T8Δ	CA-H226Y
	CA-G225D

# From ref. [51].

* In parentheses is the number of times the indicated mutation was selected.

### Replication capacity of PF-46396-resistant mutants

**Figure 5:**
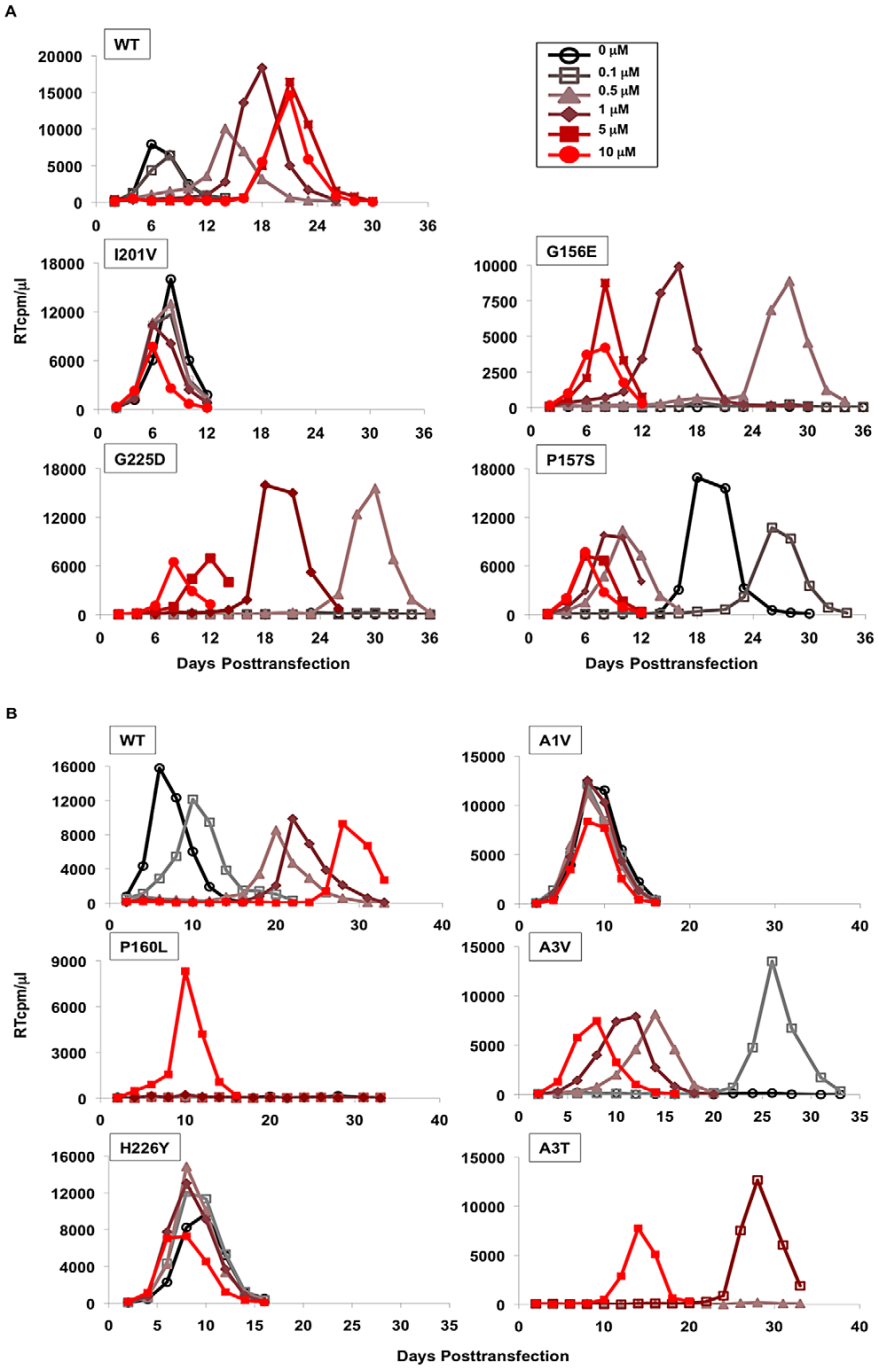
Replication kinetics of PF-46396-resistant mutants in the presence or absence of the compound. [A and B] The Jurkat T-cell line was transfected with WT or mutant pNL4-3 and propagated at the indicated concentration of PF-46396. Virus replication was monitored by RT activity, shown in cpm/µl of culture medium. Note that WT is sensitive to PF-46396; CA-I201V, CA-A1V, and CA-H226Y are resistant; and CA-G156E, CA-P157S, CA-G225D, CA-P160L, SP1-A3V and SP1-A3T are PF-46396-dependent.

We next sought to determine the replication capacity of the mutants shown in Fig. 4 and Table 1 in the presence and absence of PF-46396. Specifically, we sought to confirm that these mutations confer resistance to the compound. We also wanted to determine the effect of one of our previously described BVM resistance mutations, SP1-A1V [38], [40], on susceptibility to PF-46396. The mutations were each introduced independently into pNL4-3, and the Jurkat T-cell line was transfected with WT and mutant molecular clones. Transfected cells were propagated in the presence or absence of PF-46396 at the indicated concentrations (Fig. 5). As shown earlier (Fig. 4A), WT NL4-3 replicated with a peak at around 1 week posttransfection in the absence of compound, whereas the RT peak was delayed significantly in the presence of PF-46396, with peaks at around 3 weeks posttransfection at higher compound concentrations. Mutants CA-I201V, CA-H226Y, and SP1-A1V replicated with similar kinetics in the presence and absence of PF-46396, demonstrating that these mutants are compound-resistant. The CA MHR mutants (G156E, P157S, and P160L) and CA-G225D typically failed to replicate in the absence of PF-46396 or replicated with a significant delay relative to WT (upon acquiring second-site mutations; see below). Remarkably, however, the replication of these mutants peaked at approximately 1 week, or soon thereafter, in the presence of high concentrations of the compound. These results demonstrate that the replication of the MHR mutants and CA-G225D is compound-dependent. In some cases (e.g., CA-P157S in Fig. 5A) delayed replication in the absence of PF-46396 resulted from the acquisition of second-site compensatory mutations (see below). The A3V and A3T mutants, which we previously showed were replication defective but enhanced by BVM [40], also replicated in a PF-46396-dependent fashion (Fig. 5).

### PF-46396-dependent CA mutants display a severe defect in virus particle production in the absence of the compound

**Figure 6:**
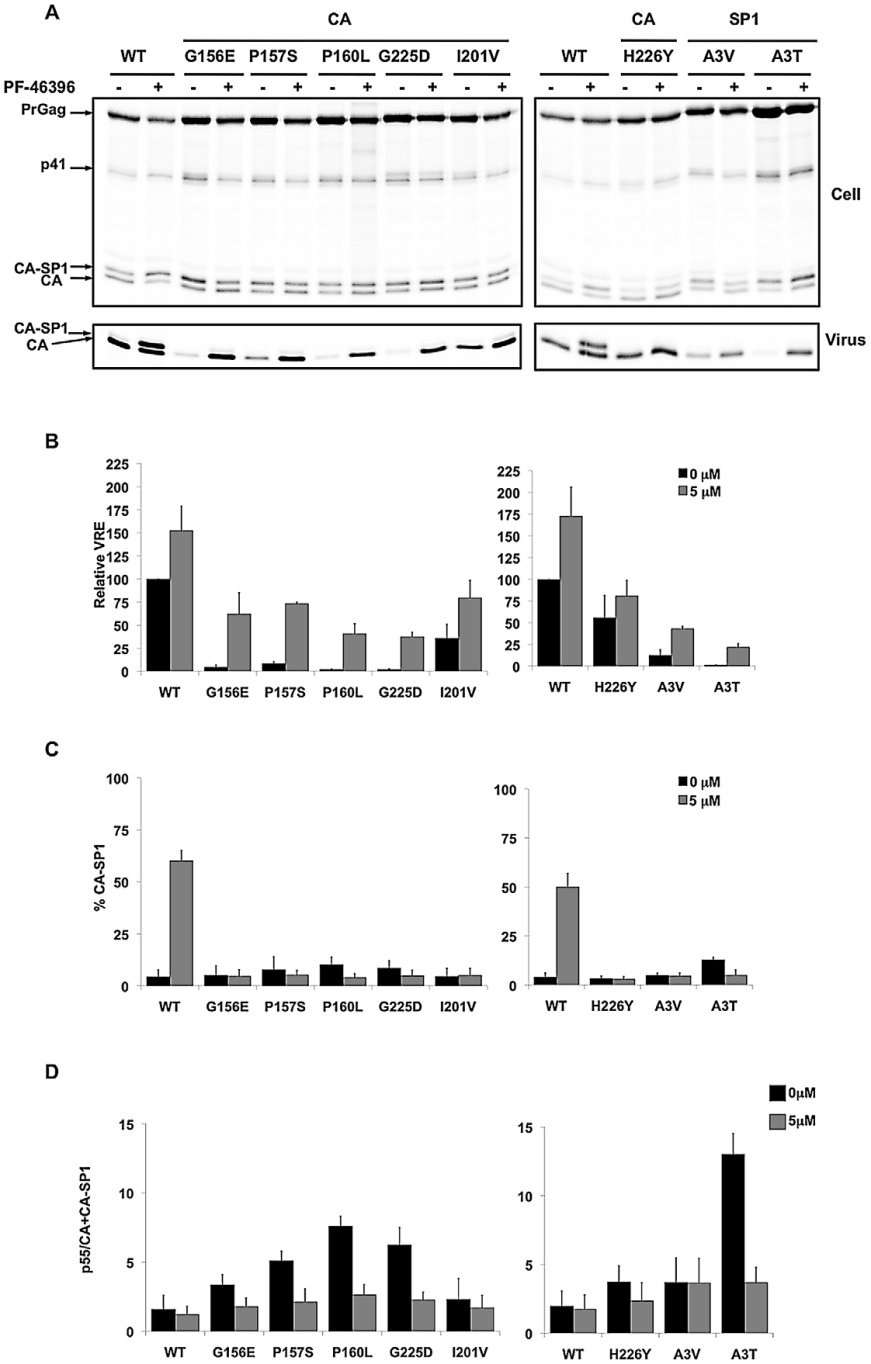
Characterization of PF-46396-resistant mutants. [A] Virus release and CA-SP1 processing in the presence or absence of PF-46396. 293T cells were transfected with WT or mutant pNL4-3 molecular clones, untreated (−) or treated (+) with 5 µM PF-463496 and metabolically labeled with [^35^S]MetCys. Cell- and virus-associated proteins were radioimunoprecipitated. Positions of Pr55^Gag^ (PrGag), Pr41^Gag^ (p41), CA-SP1 and CA are indicated. [B] Quantification of relative virus release efficiency (VRE) at the indicated concentrations of PF-46396, with release efficiency of WT in the absence of PF-46396 set at 100. Data obtained by phosphorimager analysis of radioimmunoprecipitation data. VRE is calculated as [virus-associated CA+CA-SP1]/[total (cell+virus) Gag]. Error bars indicate SD; N = 3. [C] Quantification of the % CA-SP1 relative to total CA+ CA-SP1 in virion fraction at the indicated concentrations of PF-46396. Error bars indicate SD; N = 3. [D] Quantification of the ratio of cell-associated Pr55^Gag^ (p55) to CA+CA-SP1 at the indicated concentrations of PF-46396. Data obtained by phosphorimager analysis of radioimmunoprecipitation data. Error bars denote SD; N = 3.

As mentioned in the Introduction, the MHR has been shown to play a critical role in HIV-1 assembly and release. We thus sought to examine the assembly/release properties of the compound-dependent MHR mutants (G156E, P157S, and P160L) in the presence and absence of compound. The compound-dependent but non-MHR mutant CA-G225D and the PF-46396-resistant mutant CA-I201V were also included in this analysis. 293T cells were transfected with WT or mutant HIV-1 molecular clones, treated or not with PF-46396, and metabolically radiolabeled for 2 h. Cell and viral lysates were prepared and immunoprecipitated with anti-HIV-1 antiserum (HIV-Ig) and viral proteins were visualized by SDS-PAGE and fluorography (Fig. 6A). As shown earlier in Fig. 1B for the WT, PF-46396 treatment led to the accumulation of CA-SP1 in both cell and virion fractions (quantified in Fig. 6C). PF-46396 did not have a significant effect on CA-SP1 processing for the I201V mutant, confirming the replication data indicating that this mutant is PF-46396-resistant (Fig. 6A and C; [53]). Most interestingly, the CA-G156E, P157S, P160L, and G225D mutants showed a severe defect in particle production in the absence of PF-46396 but a substantial rescue of particle assembly and release in the presence of the compound (Fig. 6A and B). For example, CA-G156E virus release efficiency was ∼4% that of the WT in the absence of compound, but ∼60% that of the WT in its presence. The defect in particle production observed for CA-G156E, P157S, P160L, G225D and SP1-A3T mutants was also associated with a modest accumulation of Pr55^Gag^ in cell-associated fractions; this defect was also corrected by the compound (Fig. 6D). CA-SP1 processing was not inhibited by PF-46396 in the context of these mutant Gags (Fig. 6C) confirming that they are PF-46396-resistant.

**Figure 7:**
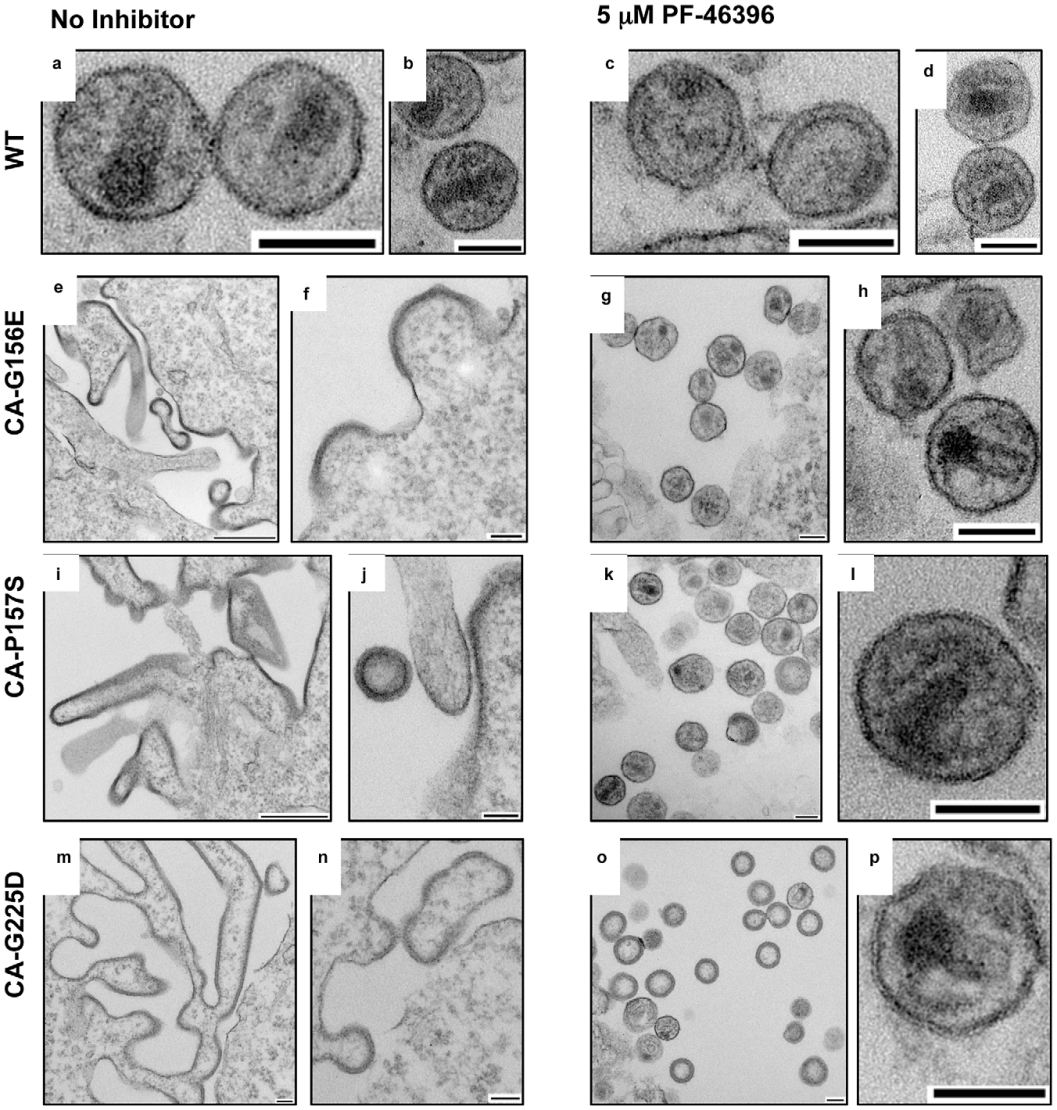
EM analysis of 293T cells transfected with WT pNL4-3 or the indicated mutant derivatives and treated without (no inhibitor) or with 5 µM PF-46396. Note highly defective EM morphology and accumulation of Gag at the plasma membrane for the mutants in the absence of PF-46396, and the rescue of assembly/release in the presence of compound. Scale bars: a–d, f–h, j–p = 100 nm; e, i = 500 nm.

The data in Fig. 6A and B demonstrate that PF-46396 rescues a severe defect in particle assembly and release imposed by the CA-G156E, P157S, and G225D mutations. To visualize this phenomenon at the particle morphogenesis level, we performed EM analysis of 293T cells transfected with WT or mutant molecular clones (Fig. 7). In the absence of PF-46396, cells transfected with CA-G156E, P157S, and G225D showed the presence of electron-dense patches of Gag at the plasma membrane, but limited evidence of particle release. In contrast, in the presence of the compound, large numbers of particles, many with mature conical cores, were released. These biochemical and EM results, together with the replication data presented in Fig. 5, establish the marked compound dependence of the MHR and CA-G225D mutations.

### The PF-46396-dependent CA mutants are not rescued by BVM

**Figure 8:**
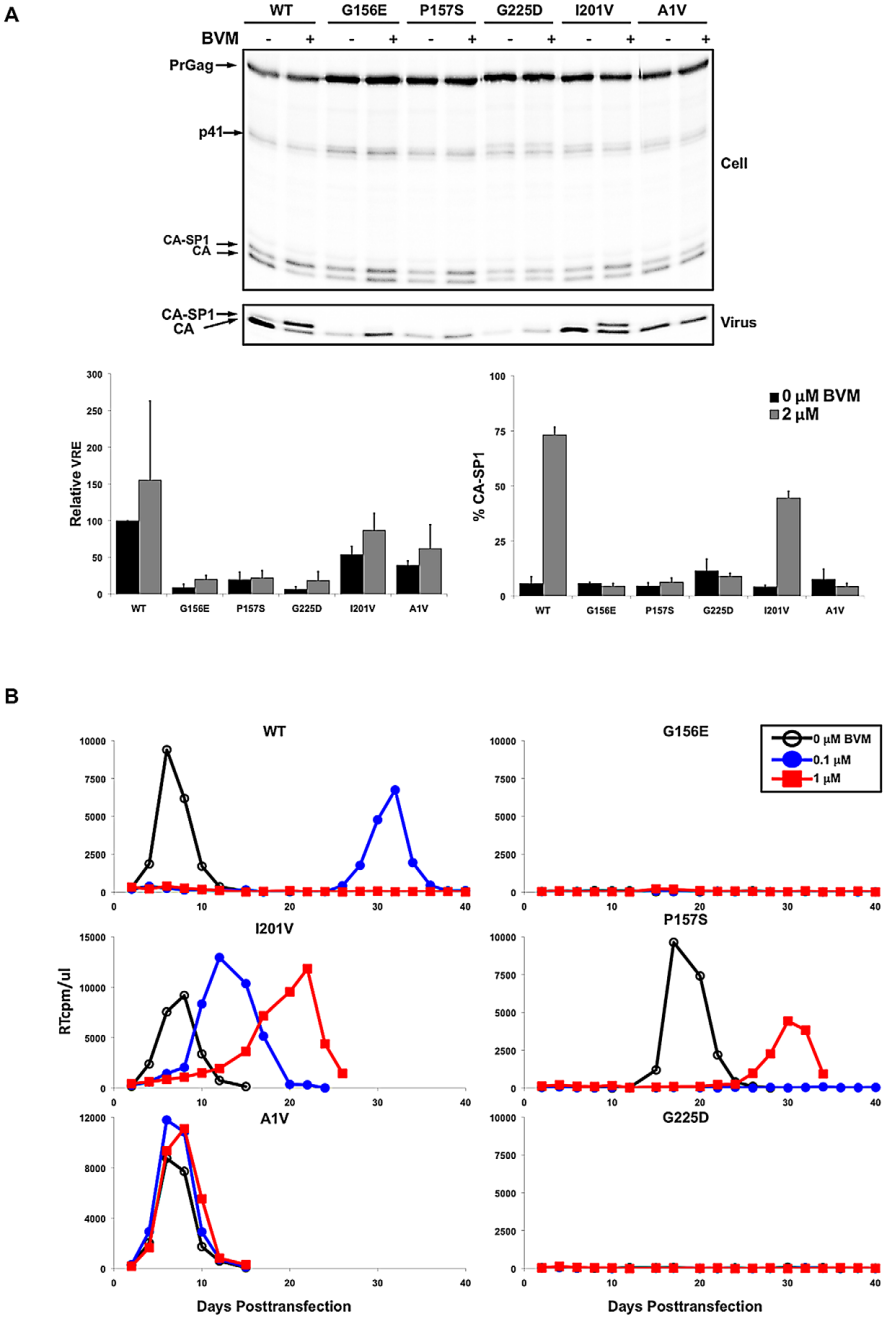
PF-46396-dependent mutants are not rescued by BVM. [A] Radioimmunoprecipitation analysis of cell- and virion-associated proteins in the absence (−) and presence (+) of 2 µM BVM. Positions of Pr55^Gag^ (PrGag), Pr41^Gag^ (p41), CA-SP1 and CA are indicated. Lower panels show phosphorimager-based quantification of relative virus release efficiency (VRE) and % virion CA-SP1, calculated as described in the Fig. 6 legend. Error bars denote SD; N = 4. [B] Virus replication kinetics in the absence and presence of BVM. The Jurkat T-cell line was transfected with WT or mutant pNL4-3 and propagated in the presence of 0, 0.1, or 1.0 µM BVM. Virus replication was monitored by RT activity, shown in cpm/µl.

The partially overlapping pattern of compound resistance observed with BVM and PF-46396 ([40] and this study) suggests that these two maturation inhibitors may share portions of a binding pocket. This, in turn, raises the question of whether BVM can rescue the virus assembly and replication defects observed with the PF-46396-dependent mutants (e.g., G156E, P157S, and G225D). To address this question, we first asked whether BVM can correct the assembly and release defects imposed by these mutations. Cells transfected with WT or mutant molecular clones were left untreated or were treated with 2 µM BVM; virus release efficiency and CA-SP1 processing were evaluated by radioimmunoprecipitation analysis as described above. In contrast to the 8 to 20-fold increase in virus release efficiency observed for these mutants in the presence of PF-46396 (Fig. 6A and B), BVM had little or no effect on their release (Fig. 8A). BVM also had no effect on CA-SP1 processing for these PF-46396-dependent mutants, likely reflecting a major change in the conformation of the compound binding pocket that prevented BVM binding (Fig. 8A; see Discussion). As expected, the previously described [38], [40] BVM-resistant mutant SP1-A1V was not affected by BVM, whereas CA-SP1 processing was disrupted for the PF-46396-resistant (but non-dependent) mutant CA-I201V (Fig. 8A).

The inability of BVM to correct the defects imposed by the CA-G156E, P157S and G225D mutations was confirmed in multiple-round replication assays (Fig. 8B). The Jurkat T-cell line was transfected with WT or mutant HIV-1 molecular clones and cells were passaged in the absence of BVM or at 0.1 or 1 µM concentrations of the compound. No enhancement was observed in the ability of these mutants to establish a spreading infection. We also tested the PF-46396-resistant but non-dependent mutant I201V; this mutant showed a partially resistant phenotype; its replication was delayed to a lesser extent than that of WT, but it was also more sensitive to BVM than the previously described [38], [40] BVM-resistant mutant SP1-A1V (Fig. 8B).

### PF-46396 can rescue defects imposed by some, but not all, MHR mutations

**Figure 9:**
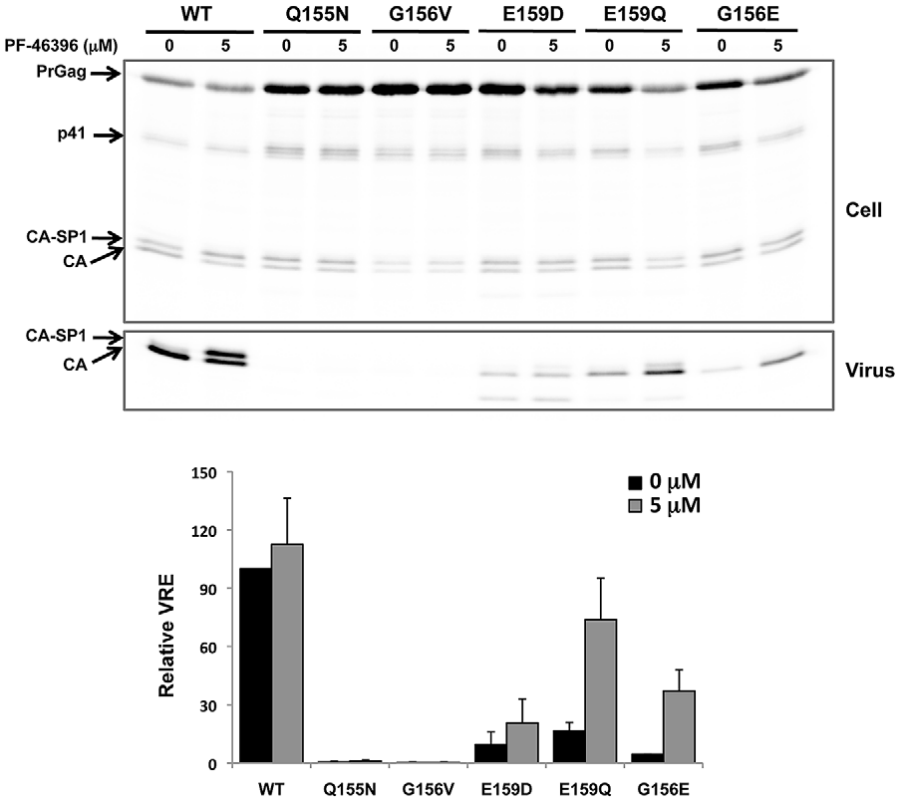
PF-46396 can rescue the assembly/release defect of some but not all MHR mutants. Top panel: Radioimmunoprecipitation analysis of cell- and virion-associated proteins in the absence and presence of 5 µM PF-46396. Positions of Pr55^Gag^ (PrGag), Pr41^Gag^ (p41), CA-SP1, and CA are indicated. Lower panel shows phosphorimager-based quantification of relative virus release efficiency (VRE), calculated as described in the Fig. 6 legend. Error bars denote SD; N = 3. Note that PF-46396 significantly enhances the release of virus particles for E159Q (and G156E, as shown earlier) but not for Q155N, G156V, or E159D.

The MHR consists of a stretch of 20 amino acids highly conserved across diverse genera of *Orthoretroviridae*. Several residues are particularly well conserved: in HIV-1 CA, these correspond to residues 155Q, 156G, 159E, and 167R (indicated with a double underline in Fig. 4B). Mutations at these positions in the MHR of HIV-1 or the analogous residues in the MHR of other retroviruses have been reported to cause severe defects in Gag processing, assembly, and release (see Introduction). Because, as described above, MHR mutants emerged during selection in PF-46396, and because these mutants showed a pronounced degree of compound dependence, we asked whether PF-46396 could also rescue the assembly defects imposed by other mutations in the MHR. To this end, we introduced the following mutations at highly conserved MHR positions: CA-Q155N, G156V, E159D, and E159Q, and tested the effects of these substitutions on particle production with and without PF-46396. The compound-dependent mutant CA-G156E was included in these assays as a control. As expected, all mutations severely affected virus assembly and release (Fig. 9), with the CA-Q155N and G156V essentially abolishing detectable virus particle production and the G156E, E159D, E159Q mutations reducing virus production by ∼10–20 fold relative to that of the WT. Addition of 5 µM PF-46396 to the producer cells markedly enhanced particle production for CA-G156E (as shown above) but failed to rescue particle assembly in the case of the most severely affected mutants (Q155N and G156V). A small and statistically insignificant increase in particle release was observed with the E159D mutant. In contrast, PF-46396 was able to increase particle production by ∼4-fold for the E159Q mutant (Fig. 9). As we observed for the MHR mutants selected during passaging in the compound, PF-46396 had little or no effect on CA-SP1 processing for these mutants relative to that observed for the WT. These results indicate that PF-46396 is able to rescue the assembly/release defects imposed by a subset of mutations in the CA MHR.

### Second-site compensatory changes rescue the defects imposed by PF-46396-dependent mutants

The PF-46396-dependent mutants showed delayed replication in the absence or at low concentrations of PF-46396 (Fig. 5). To evaluate whether this delayed replication was associated with the acquisition of second-site compensatory mutations, we PCR amplified viral DNA from infected cultures at peak RT activity and performed DNA sequencing. Table 2 indicates the second-site changes that arose upon passaging of the CA-G156E, P157S, and G225D in the absence or presence of low concentrations of the compound. The CA-G156E mutant acquired a CA-N193H change. The CA-P157S mutant obtained a CA-G225S or an SP1-T8I change. The CA-G225D mutant also acquired an SP1-T8I substitution, or reverted back to an Asn at residue 225 (CA-G225N). It is interesting to note that we previously reported that CA-G225S acts as a compensatory mutation for SP1-A3V [40].

**Table 2:** Second-site compensatory changes for PF-46396-dependent CA mutants.

First Mutation	PF-46396 concentration	Second-site change
CA-G156E	0.5 µM	CA-N193H
CA-P157S	0 µM	SP1-T8I
	0.1 µM	SP1-T8I
		CA-G225S
CA-G225D	0.5 µM	SP1-T8I
	1 µM	SP1-T8I
		CA-G225N

**Figure 10:**
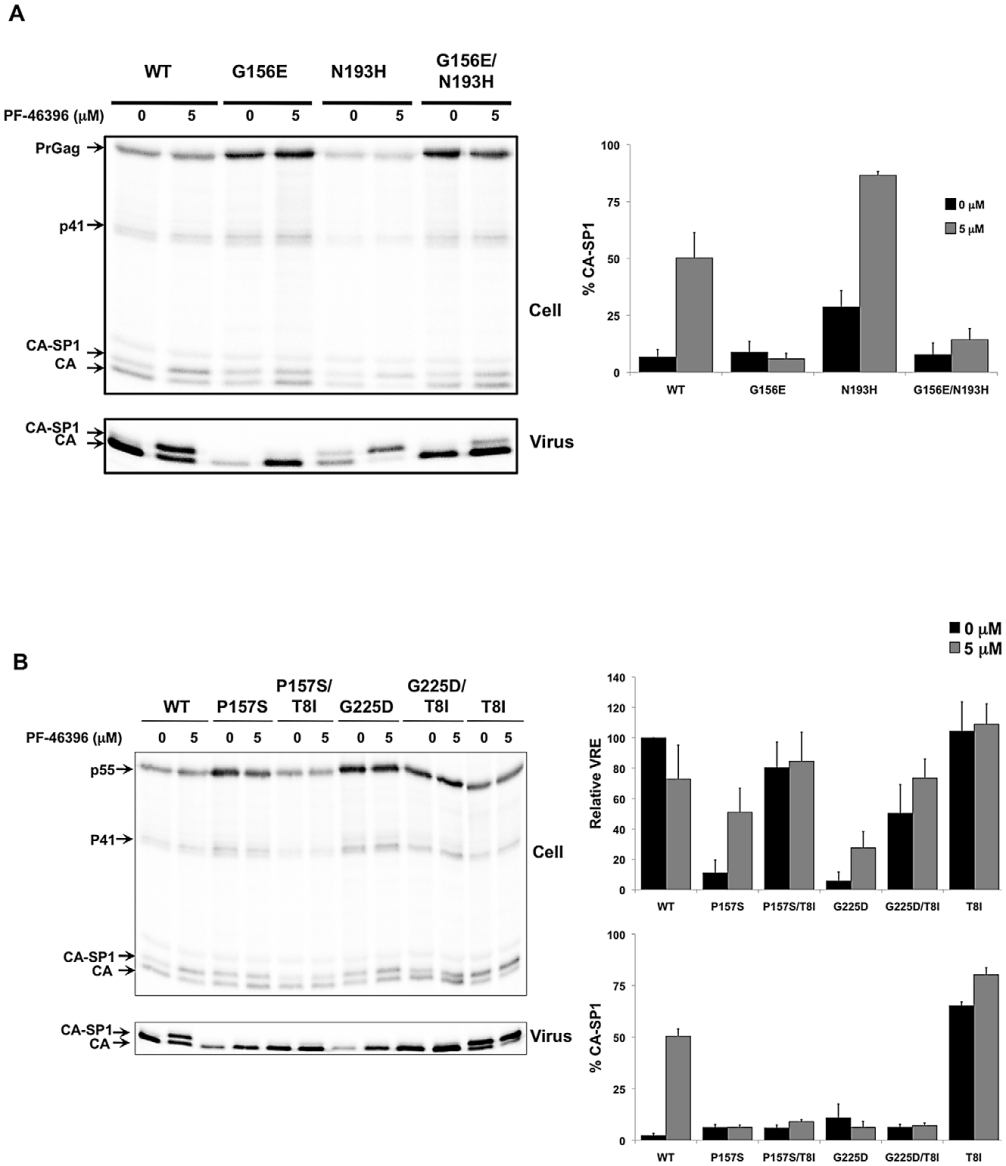
Second-site compensatory changes correct the replication defects exhibited by PF-46396-dependent CA mutants. [A] Radioimmunoprecipitation analysis of cell- and virion-associated proteins in the absence and presence of 5 µM PF-46396. Analysis performed with WT, CA-G156E, CA-N193H, and CA-G156E/N193H. Positions of Pr55^Gag^ (PrGag), Pr41^Gag^ (p41), CA-SP1, and CA are indicated. Graph on the right shows phosphorimager-based quantification of the % CA-SP1 relative to total CA+ CA-SP1 in virion fraction at the indicated concentration of PF-46396. Error bars denote SD; N = 5. [B] Radioimmunoprecipitation analysis of cell- and virion-associated proteins in the absence and presence of 5 µM PF-46396. Analysis performed with WT, CA-G157S, CA-P157S/SP1-T8I, CA-G225D, CA-G225D/SP1-T8I, and SP1-T8I. Positions of Pr55^Gag^ (PrGag), Pr41^Gag^ (p41), CA-SP1, and CA are indicated. Graphs on the right show phosphorimager-based quantification of relative virus release efficiency (VRE) and % virion CA-SP1, calculated as described in the Fig. 6 legend. Error bars denote SD; N = 3.

To assess whether the second-site mutations could rescue the assembly and replication defects exhibited by the PF-46396-dependent mutants, we constructed G156E/N193H, P157S/T8I and G225D/T8I double mutants and carried out virus release and replication assays. We also evaluated the phenotype of the second-site mutants N193H and T8I alone. The CA-N193H mutation by itself did not affect virus release efficiency, but interestingly this mutant was more sensitive to PF-46396-mediated inhibition of CA-SP1 processing than the WT, with approximately 90% accumulation of CA-SP1 at 5 µM of the compound (Fig. 10A). The G156E/N193H double mutant displayed a several-fold increase in virus release efficiency relative to that of the G156E single mutant, but was still released with an efficiency of only ∼30% that of the WT (Fig. 10A; data not shown). Analysis of the T8I single and double mutants (Fig. 10B) demonstrated that T8I was able to markedly rescue the assembly/release defect observed with the CA-P157S and G225D single mutants. The double mutants remained insensitive to PF-46396 in terms of CA-SP1 processing. The T8I single mutant was released efficiently but, interestingly, showed a high level of CA-SP1 accumulation even in the absence of PF-46396.

**Figure 11:**
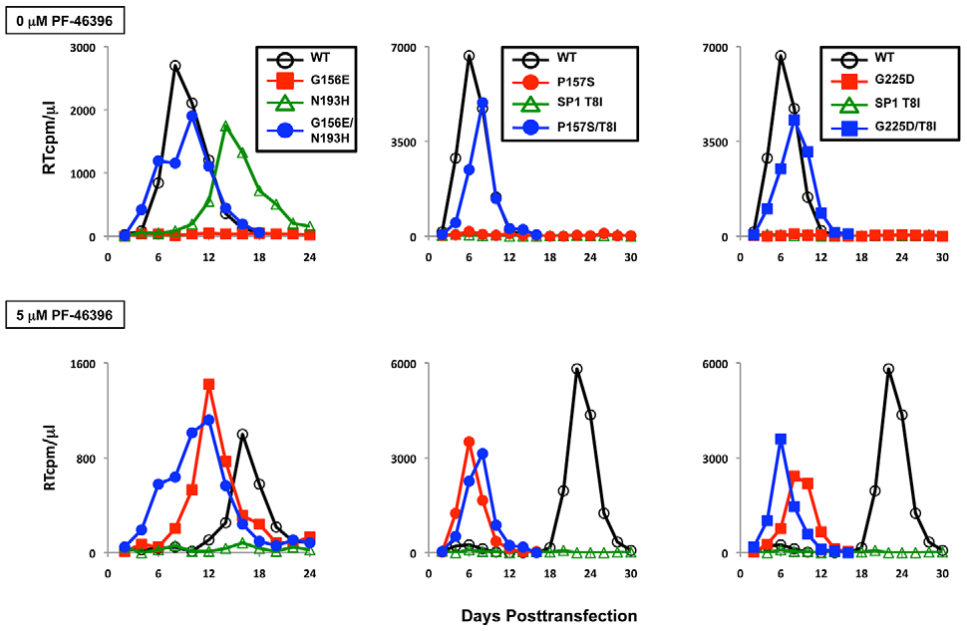
Virus replication kinetics in the absence (top) and presence (bottom) of PF-46396. The Jurkat T-cell line was transfected with WT or mutant pNL4-3 and propagated in the presence of 0 or 5 µM PF-46396. Virus replication was monitored by RT activity, shown in cpm/µl.

In virus replication assays in Jurkat, the second-site mutations were able to rescue the replication defects imposed by the original PF-46396-dependent mutations (Fig. 11). As shown above (Fig. 5A), G156E, P157S, and G225D were replication defective in the absence of PF-46396 but replicated in its presence (Fig. 11). The G156E/N193H, P157S/T8I and G225D/T8I double mutants were replication competent and compound-resistant. The N193H single mutant showed a ∼1 wk delay in peak replication relative to the WT and was sensitive to PF-46396, consistent with the CA-SP1 processing data (Fig. 10A). In most experiments, SP1-T8I was replication deficient, as one might predict from the high level of CA-SP1 accumulation seen with this mutant. In one experiment, T8I replication was observed in the absence of inhibitor, with a peak of RT activity around day 20 postinfection, presumably reflecting the acquisition of additional change(s) (data not shown). The P157S/G225S double mutant replicated with near-WT kinetics and was PF-46396-resistant (data not shown). Together, these data demonstrate that second-site mutations acquired during propagation of the PF-46396-dependent mutants are able to compensate for the replication defects observed for these mutants. The resulting viruses are both replication-competent and compound-resistant. It is significant that these compensatory changes emerged in the same three domains to which compound resistance mapped (i.e., the CA MHR, in the vicinity of CA residue 200, and SP1), suggesting structural and/or functional cross-talk between these three regions of Gag (see Discussion).

### The PF-46396-dependent MHR mutants exhibit a defect in Gag multimerization

The above-described PF-46396-dependent MHR mutants display a severe defect in virus particle production that is rescued by second-site compensatory changes far downstream in Gag (e.g., SP1-T8I). To understand which step(s) in the assembly/release pathway are disrupted by these mutations, we examined *in vivo* Gag multimerization and Gag-membrane binding. Membrane flotation centrifugation assays were performed to evaluate the percentage of Gag that is associated with membrane in virus-producing 293T cells. As a control, we used the non-myristylated Gag mutant 1GA [55]. Glyceraldehyde 3-phosphate dehydrogenase (GAPDH) served as a non-membrane-associated control protein. As we reported previously [56], [57], [58], we observed that approximately 50% of WT Gag is present in membrane fractions (Fig. S2). As expected, GAPDH was located exclusively in the bottom (non-membrane) fractions. The non-myristylated 1GA Gag mutant showed minimal membrane association (3% of Gag in membrane fractions). The CA-G156E and P157S mutants showed reductions in membrane association, but these reductions were not highly significant. As expected given their WT or near-WT virus release efficiency (Fig. 10A) the P157S/T8I and T8I mutants showed WT levels of Gag-membrane association (data not shown). We next evaluated these mutants for their ability to undergo multimerization in cells, using our previously reported assay in which Gag multimerization is measured by comparing the amount of Gag immunoprecipitated without denaturation (in which highly assembled Gag is recognized inefficiently by anti-Gag antibodies) vs. the amount of Gag recognized after denaturation ([59]; Materials and Methods). Again, non-myristylated 1GA Gag, which does not assemble into higher-order Gag multimers that undergo antibody epitope masking, was used as a control. Under the conditions of this analysis, approximately 60% of WT Gag had undergone higher-order Gag multimerization, whereas only ∼20% of 1GA Gag was epitope masked (Fig. S3). The CA-G156E and P157S showed defects in Gag multimerization that were comparable to those of 1GA. The assembly competent revertant, P157S/T8I, showed WT levels of Gag multimerization, whereas the T8I single mutant displayed a small but statistically significant increase in assembly. These results indicate that the primary defect in particle production for the MHR mutants is exerted at the level of Gag-Gag multimerization.

### PF-46396-dependent mutants can be triggered to assemble with compound

**Figure 12:**
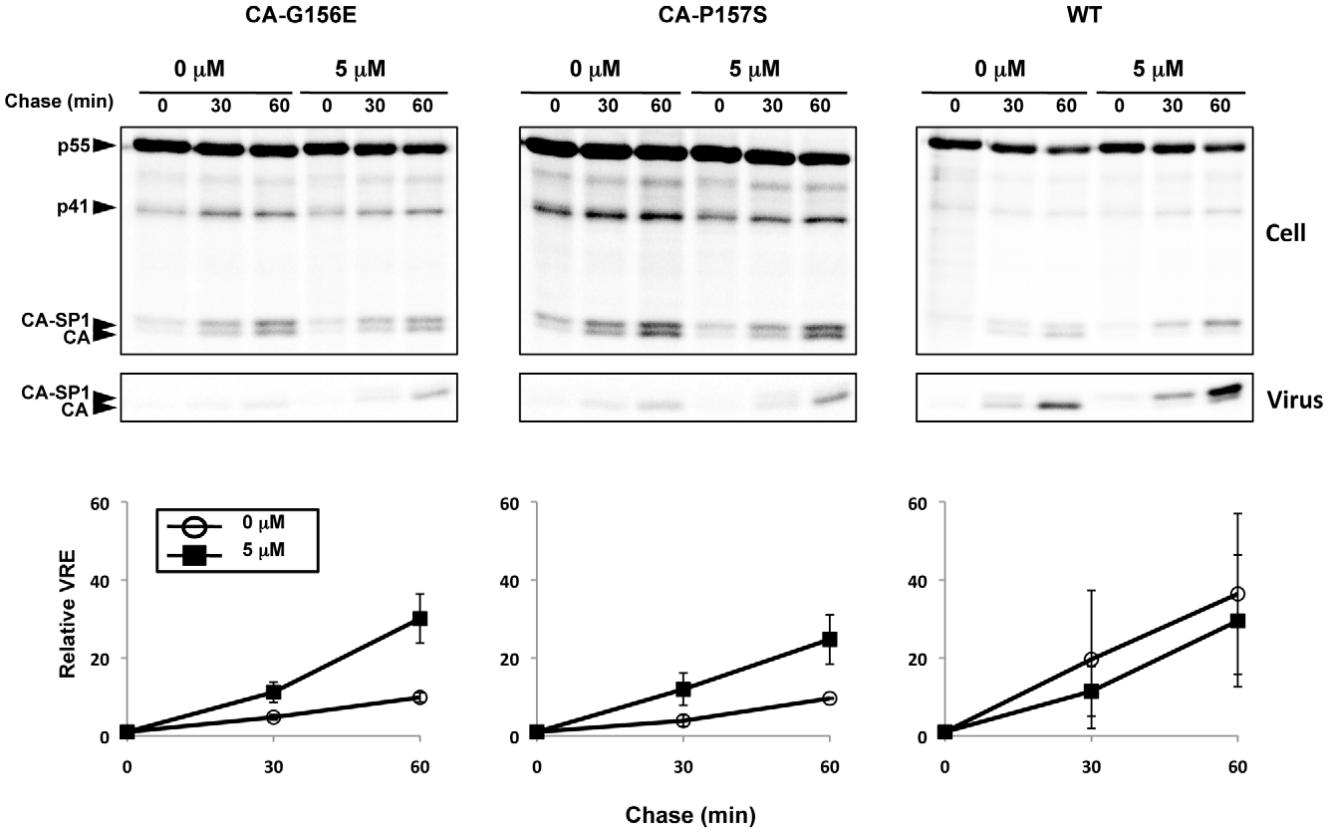
PF-46396 can trigger the release of compound-dependent MHR mutants. 293T cells were transfected with WT or mutant pNL4-3 molecular clones. At 24 h posttransfection, cells were starved for 30 min in Met/Cys-free medium and metabolically labeled with [^35^S]Met/Cys for 20 min. Cells were divided into two fractions, washed, and resuspended in medium containing no or 5 µM PF-46396. Each fraction was further divided into three aliquots and incubated at 37°C for the indicated chase times. Cell and viral lysates were radioimmunoprecipitated. Positions of Pr55^Gag^ (PrGag), Pr41^Gag^ (p41), CA-SP1, and CA are indicated. Graphs in the lower panels show phosphorimager-based quantification of relative virus release efficiency (VRE) calculated as described in the Fig. 6 legend. Error bars indicate SD; N = 3.

The data presented in Fig. S3 demonstrate that the PF-46396-dependent mutants are defective for Gag oligomerization, presumably due to disruptions in Gag folding induced by the mutations. The presence of PF-46396 during the entire time course of the analysis (from transfection through metabolic radiolabeling) is able to rescue this assembly defect (e.g., Fig. 6). To determine whether comparatively brief exposure of these compound-dependent mutants to PF-46396 could trigger particle assembly and release, we examined the kinetics with which this defect could be reversed by PF-46396. Cells were transfected with WT, CA-G156E or CA-P157S molecular clones then pulse-labeled with [^35^S]Met/Cys. Cells were then chased for 0, 30, or 60 min in cold medium containing 0 or 5 µM PF-46396. At the end of the chase time, cell and viral lysates were prepared and analyzed as described above. In WT-transfected cultures virus release efficiency did not differ significantly between PF-46396-treated and untreated cultures (Fig. 12). In contrast, even at the 30 min chase time point, significantly more virus was detected in PF-46396-treated vs. untreated cultures. The difference between treated and untreated cultures increased at the 60 min time point. These data demonstrate that addition of PF-46396 rapidly triggers the assembly and release of the assembly-deficient MHR mutants.

## Discussion

**Figure 13:**
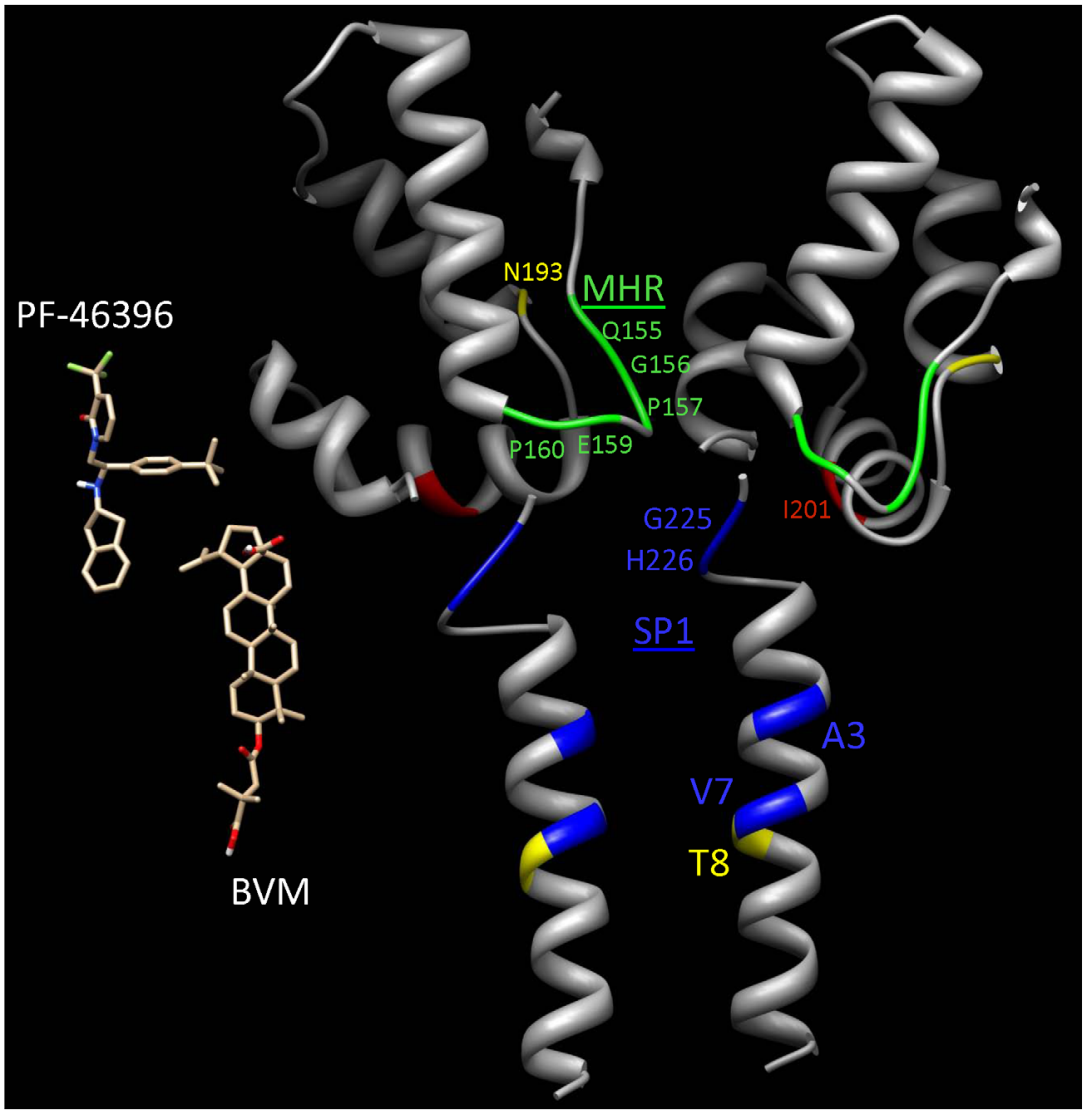
Schematic molecular model of PF-46396 and BVM binding sites. The ribbon diagrams illustrate two adjacent CA-CTD/SP1 monomers in the hexagonal complex of the immature capsid. The atomic coordinates of the CA-CTD and SP1 domains were obtained from PDB 3H4E [66] and PDB 1U57 [27], respectively. Residues 220–223 that link the CA-CTD and SP1 domains of each monomer were not modeled due to a lack of experimental data. The relative orientations of the adjacent CA-CTD domains were taken from the models of Bharat et al. [61]. The sites of resistance mutations are labeled and color-coded for location: Green, blue and red for the MHR, CA-CTD/SP1 boundary, and individual I201 residue, respectively. Yellow indicates the sites of secondary substitutions that rescue the G156E and P157S mutants. Also shown are stick figures of the PF-46396 and BVM compounds at the heights of their respective binding sites predicted from the locations of the resistance mutations (color-code: C – brown, N - blue, O - red, H – white, and Fl – green). The rotational orientation of PF-46396 is arbitrary, but that of BVM is taken from the photoafinity data of Nguyen et al. [45]. The proximity of the MHR of the left monomer to the CA-CTD/SP1 region of the right one suggests that the binding sites of the two compounds straddle adjacent monomers. This would explain the observed necessity of Gag assembly into the immature capsid structure for BVM cleavage inhibition (see text).

In this study, we demonstrate that resistance to PF-46396 maps to three domains in Gag: the CA-SP1 boundary region, CA residue 201, and the CA MHR. Several of the resistance mutations in the CA-SP1 boundary region were observed in our previous studies with BVM; however, resistance mutations in the MHR or CA-I201 were not acquired during extensive selections in BVM. These results suggest that although BVM and PF-46396 likely share portions of a binding pocket, distinct upstream contacts are made by PF-46396 (see structure model, Fig. 13). The proximity of the MHR, CA residue 201, and the CA-SP1 boundary region is suggested by structural models (e.g., [28] in which the MHR and residue 201 are located near the base of the CA CTD from which projects the putative helical bundle of SP1 peptides (see Fig. 13). The functional cross-talk between these three regions is demonstrated by our observation that second-site changes that rescue the assembly and replication defect of MHR mutants (G156E and P157S) map to residue 193 of CA (CA-N193H) or residue 8 of SP1 (SP1-T8I). Although MHR mutations arose repeatedly during selection in PF-46396 but never during selection in BVM [40], in experiments using a BVM analog with a photoaffinity label, some compound cross-linking was detected at the MHR [45]. These results suggest that BVM engages in contacts, albeit perhaps transient or low affinity, with the MHR. The inability of BVM to rescue the replication defects imposed by the MHR mutations could explain why MHR mutations were never observed during selections in BVM. It is interesting to note that in the avian retrovirus system (Rous sarcoma virus), mutations in the spacer peptide downstream of the CA domain was reported to rescue defects imposed by MHR changes [18]. NMR analysis of a fragment of Rous sarcoma virus Gag spanning the CA CTD, the SP, and NC also provided evidence for an interaction between the MHR and SP [60]. These results, coupled with our findings, suggest that interactions between the MHR in CA and downstream putatively helical domains (SP1 in the case of HIV-1, SP in the case of Rous sarcoma virus) may be a general feature of orthoretroviral Gag assembly. The ability of PF-46396 to block the activity of low concentrations of BVM is consistent with a partially shared binding pocket, though a mechanism of allosteric antagonism cannot be excluded at this time.

As discussed in the Introduction, naturally occurring polymorphisms in SP1, most notably SP1-V7A, significantly reduce the ability of BVM to disrupt CA-SP1 processing [48], [50], [51]. It was therefore of interest to test whether changes such as V7A likewise interfere with the activity of PF-46396. Although its potency is lower than that of BVM, we observed that PF-46396 is less sensitive to the V7A change relative to BVM. A simple model to explain this finding is that SP1 residue 7 comprises part of the BVM but not PF-46396 binding site (Fig. 13). Alternatively, the SP1-V7A polymorphism could alter the conformation of the maturation inhibitor binding site such that the binding or activity of BVM but not PF-46396 is compromised. In any case, the relative lack of sensitivity of PF-46396 to SP1-V7A suggests that maturation inhibitors can be developed that are active against a range of strains containing polymorphisms in SP1. However, it should be noted that although QVT polymorphisms appear to have little effect on sensitivity to PF-46396, Blair and colleagues [53] observed that a number of primary clinical isolates were relatively refractory to inhibition by the compound. The genetic basis for this insensitivity awaits further study.

A notable feature of several PF-46396-resistant mutants (CA-G156E, P157S, P160L, and G225E) is their high level of compound dependence. Most of these changes fall within the CA MHR, a 20-amino-acid sequence highly conserved among retroviral Gag proteins (see Introduction). In the absence of PF-46396, the MHR mutants that arose during PF-46396 selection are severely deficient for virus particle production. Biochemical assays demonstrate that these mutants are primarily deficient in Gag multimerization. We propose that binding of PF-46396 rescues these multimerization defects, possibly by correcting the impaired folding induced by the mutations. This rescue is specific to PF-46396, as BVM exerts little or no effect on virus particle production for these MHR mutants and does not rescue their replication. We observe that defective Gag can be triggered to release following relatively short PF-46396 treatments. This compound-induced release may have useful applications in a variety of HIV-1 assembly analyses; for example, in imaging studies.

Based on the model presented in Fig. 13, it is possible that PF-46396 could make contacts with the CA-SP1 boundary region, residue 201, and the MHR. Because structural studies suggest that these regions of Gag are close to each other in the assembled Gag complex (e.g., [28], [61]), in our view this is the most straightforward model that is consistent with available data, including recent BVM binding results [45]. However, alternative models cannot be excluded at this time. For example, it is possible that the relationship between the MHR and maturation inhibitors is indirect. PF-46396 could stabilize the Gag multimer, thereby limiting the ability of PR to access and cleave the CA-SP1 junction. In such an allosteric model, mutations in the MHR would destabilize the Gag multimer, thereby reversing the effect of the compound on multimer stability. The opposing effect on Gag multimerization of MHR mutations and PF-46396 binding could explain the phenomenon of compound dependence. Support for the hypothesis that maturation inhibitors can stabilize the immature Gag lattice is provided by recent cryo-EM findings [54]. Additional details regarding the structure of the MHR and SP1 in the context of assembled Gag, and more information about the residues that engage in direct contacts with maturation inhibitors, will be required to better understand the molecular basis for maturation inhibitor activity and for the compound dependence observed in this study.

Previous studies with BVM indicated that the ability of this compound to block CA-SP1 processing requires Gag assembly [38], [44]. Furthermore, Zhou and colleagues demonstrated that BVM interacts with immature HIV-1 particles, but not with mature virions [42]. These observations are consistent with the idea that Gag assembly creates the maturation inhibitor binding site and that cleavage at the CA-SP1 junction, or at other Gag processing sites, destroys the binding pocket. Recent insights into the structure of the immature retroviral Gag lattice [61] allow one to propose that the putative maturation inhibitor-binding pocket may straddle adjacent subunits in the immature Gag lattice (Fig. 13). Such a model would explain why PR-mediated processing of monomeric Gag is not disrupted by maturation inhibitors, and why Gag processing during maturation prevents compound binding.

Although the SP1-T8I mutation rescues the replication defect imposed by two highly defective, PF-46396-dependent mutants (CA-P157S and CA-G225D) by itself the SP1-T8I mutant is severely impaired. SP1-T8I displays a high level of CA-SP1 accumulation even in the absence of maturation inhibitor and is highly replication defective, suggesting that the T8I mutation mimics the effect of PF-46396 both in its ability to block CA-SP1 processing and to rescue the assembly defects elicited by upstream mutations in CA (i.e., CA-P157S and CA-G225D).

The results presented here help to elucidate the structure-function relationship between the CA CTD and SP1, two domains of Gag that are critical to both assembly and maturation. This study also provides insights into Gag residues that comprise the HIV-1 maturation inhibitor-binding pocket. This information should be useful in the rational design of maturation inhibitors with increased potency and breadth of activity.

## Materials and Methods

### Maturation inhibitors, cell culture, and plasmids

BVM was prepared as described previously [35] and used at the concentrations indicated. Lyophilized PF-46396 (Pfizer) was suspended in DMSO to generate 10 or 20 mM stock solutions, stored at −20°C, and diluted in culture medium to the concentrations indicated. The Jurkat T-cell line was maintained in RPMI-1640 medium supplemented with 10% (vol/vol) fetal bovine serum (FBS), L-glutamine, penicillin and streptomycin. 293T cells were maintained in Dulbecco's modified Eagle's medium (DMEM) supplemented with 10% (vol/vol) FBS and L-glutamine. Plasmid DNAs were purified with the Qiagen maxiprep kit. Jurkat and 293T cells were transfected with DEAE/dextran and linear polyethylenimine (L-PEI), respectively [62], [63].

### Virus replication assays and selection for resistant mutants

PF-46396 resistance mutations were selected by multi-cycle replication assay using Jurkat T cells transfected with the WT HIV-1 molecular clone, pNL4-3 [64], in the presence of 0.5, 1.0, 5.0, and 10 µM PF-46396. Virus replication was examined by RT activity as previously described [65]. To identify resistance mutations, cell pellets were collected on the days of peak RT activity. Genomic DNA was extracted by using the whole-blood DNA purification kit (Qiagen), and the entire Gag-PR-coding regions was amplified by PCR using the primers: NL561F (5′-TGCCCGTCTGTTGTGTGACTC-3′) and NL2897R (5′-AAAATATGCATCGCCCATA-3′) [40]. The 2.3kb PCR products were purified by ExoSap-IT (Affymetrix) and sequenced using the primers: NL645F(5′-AACAGGGACTTGAAAGCGA-3′), NL1155F (5′-AGGAAACAACAGCCAGgtc-3′), NL1410F (5′-GGAAGCTGCAGAATGGGATA-3′), and NL2135F (5′-TTCAGAGCAGACCAGAGCCAA-3′). Selection for compensatory mutations to the PF-46396-dependent mutations was carried out as described above [40].

### Site-directed mutagenesis

The 4.2 kbp Spe I-Sal I fragment from pNL4-3 (nucleotide 1507 to 5785) was subcloned into Bluescript SK(+) [pBS(NL)] and mutagenized to generate CA-G156E, CA-G156V, CA-G157S, CA-E159D, CA-E159Q, CA-P160L, CA-N193H, CA-I201V, CA-G225D, CA-G225N, and SP1-T8I mutations. To generate double mutants, CA-G156E, CA-P157S, SP1-T8I clones were subjected to a second round of mutagenesis using pBS(NL)CA-N193H, pBS(NL)SP1-T8I, pBS(NL)CA-G225D subclones to generate pBS(NL)CA-G156E/CA-N193H, pBS(NL)CA-P157S/SP1-T8I, pBS(NL)CA-G225D/SP1-T8I, respectively. To generate pBS(NL)CA-P157S/CA-G225S, the 0.5 kb SpeI-ApaI fragment from pNL4-3 CA-G225S [40] was subcloned to pBS(NL) and mutagenized to generate CA-P157S/CA-G225S. All mutagenesis was performed using the QuikChange site-directed mutagenesis kit (Agilent Technologies, Santa Clara, CA). Following sequence confirmation, the SpeI-SbfI fragment was cloned back into the WT pNL4-3 to create the molecular clones containing the mutation(s) described above, which were reconfirmed by DNA sequencing.

### Radioimmunoprecipitation analysis

Radioimmunoprecipitation assays were carried out with some modification of the protocol described in detail previously [62]. Briefly, 293T cells were transfected with WT or mutant pNL4-3 by using L-PEI (4 µg L-PEI/µg DNA). At 24 hour posttransfection, HIV-1-expressing cells were starved in [^35^S]Met/Cys-free medium for 30 min and metabolically labeled with [^35^S]Met/Cys-Pro-mix (Amersham) for 2 h. Maturation inhibitors were maintained throughout the transfection and labeling. Viruses were collected by centrifugation at 99,000× g for 45–60 min. Cell and virus lysates were heated in the presence of Laemmli sample buffer (LSB) (5.7 µl 2× LSB/100 µl lysates), followed by pre-absorption for 2 h at 4°C, and immunoprecipitated with pooled immunoglobulin from HIV-1-infected patients (HIV Ig) obtained from the NIH AIDS Research and Reference Reagent Program. Immunoprecipitated proteins were separated on 13.5% acrylamide gels by SDS-PAGE, exposed to a phosphorimager plate (Fuji) and quantified by Quantity One software (Bio-Rad).

### Transmission EM

293T cells were transfected with WT or mutant pNL4-3 in the absence or presence of 5 µM PF-46396 or 2 µM BVM. Fixation of cells, preparation of samples, and transmission EM were performed as previously described [62].

### Gag multimerization assay

The Gag multimerization assay was performed with some modifications of the previously described methods [59], [62]. Briefly, 293T cells were transfected with WT or mutant pNL4-3/PR^−^ molecular clones. At 24 h posttransfection, cells were metabolically labeled with [^35^S]Met/Cys-Pro-mix for 2 h after 30 min starvation. Cells were lysed with 2× radioimmunoprecipitation assay (RIPA) buffer. Two 100 µl cell lysate aliquots were prepared: one for boiling with 2× LSB to denature Gag multimers, and the other without boiling, followed by pre-absorption, immunoprecipitation with HIV-Ig, and separation by SDS-PAGE. Gag bands were quantified by phosphorimager analysis. The extent of Gag multimerization was determined by calculating the ratio of Gag immunoprecipitated with and without sample boiling.

### Pulse-chase assay for synchronous assembly

293T cells were transfected with WT or mutant pNL4-3 molecular clones. At 24 h posttransfection, cells were starved in Met/Cys-free medium for 30 min then metabolically labeled with [^35^S]Met/Cys-Pro-mix for 20 min. Cells were divided into two fractions, washed, and resuspended in 10% FBS/DMEM containing 0 or 5 µM PF-46396. Each fraction was further divided into three aliquots and incubated at 37°C. Cells were collected at 0, 30, and 60 min chase time points. Cell lysates were immunoprecipitated with HIV-Ig and analyzed as described above.

### Membrane flotation centrifugation

293T cells were transfected with WT or mutant PR-defective (pNL4-3/PR^−^) molecular clones. Membrane flotation assays were performed as previously described [62]. Briefly, at 24 h posttransfection, cells were disrupted by sonication and post-nuclear supernatants were collected. Sonicated samples (160 µl) were mixed with 0.8 ml of 85.5% sucrose in a centrifuge tube and overlayed with 2.4 ml of 65% sucrose and 0.8 ml of 10% sucrose. Samples were subjected to ultracentrifugation at ∼100,000× g for >16 h at 4°C. Ten fractions of 0.4 ml each were collected from the top of each tube, and mixed with 0.4 ml 2× RIPA buffer. Lysates were analyzed by western blotting using HIV-Ig and anti-glyceraldehyde 3-phosphate dehydrogenase (GAPDH) (Santa Cruz Biotechnology). Gag bands were quantified by using a BioRad ChemiDoc XRS+ imaging system with Imaging Lab software.

## Supporting Information

Figure S1 Morphology of virions produced from pNL4-3-transfected 293T cells in the absence of inhibitor (a), in the presence of 2 µM BVM (b), or 5 µM PF-46396 (c–g) by thin section transmission EM. Scale bar, 100 nm. Mature, conical cores are produced in the absence of inhibitor (black arrow). In the presence of maturation inhibitor, particles are observed to contain a crescent of electron density (white arrow) and a condensed aggregate of electron-dense material (red arrow). Particles produced from PF-46396 display greater morphological heterogeneity, including particles that contain both the electron-dense crescent and a condensed, conical core-like structure (blue arrow).
(TIF)

Figure S2 Membrane binding analysis for CA-G156E and P157S mutants. 293T cells were transfected with WT or mutant PR-defective (pNL4-3/PR^−^) molecular clones. Membrane flotation assays were performed as described in the Materials and Methods. Gag and GAPDH were detected by quantitative western blotting; bands were quantified by Imaging Lab software (Bio-Rad). WT and non-myristylated Gag (1GA) serve as positive and negative controls, respectively. P values: **, p<0.01; *, p<0.05. Dashed line indicates lack of statistical significance. N = 2–3. Frx = fraction.
(TIF)

Figure S3 PF-46396-dependent MHR mutants are defective for Gag multimerization. Gag multimerization was evaluated in a cell-based assay described in the Materials and Methods. WT and the non-myristylated Gag mutant (1GA) served as positive and negative controls, respectively. P values: **, p<0.01; *, p<0.05. Note that the second-site compensatory mutation SP1-T8I rescues the multimerization defect imposed by the CA-P157S mutation. Error bars indicate SD; N = 3.
(TIF)

## References

[1] Gulick RM, Mellors JW, Havlir D, Eron JJ, Gonzalez C, et al (1997) Treatment with indinavir, zidovudine, and lamivudine in adults with human immunodeficiency virus infection and prior antiretroviral therapy. N Engl J Med 337: 734–739. 9287228 10.1056/NEJM199709113371102

[2] Hammer SM, Squires KE, Hughes MD, Grimes JM, Demeter LM, et al (1997) A controlled trial of two nucleoside analogues plus indinavir in persons with human immunodeficiency virus infection and CD4 cell counts of 200 per cubic millimeter or less. AIDS Clinical Trials Group 320 Study Team. N Engl J Med 337: 725–733. 9287227 10.1056/NEJM199709113371101

[3] Richman DD (2001) HIV chemotherapy. Nature 410: 995–1001. 11309630 10.1038/35073673

[4] Walensky RP, Paltiel AD, Losina E, Mercincavage LM, Schackman BR, et al (2006) The survival benefits of AIDS treatment in the United States. J Infect Dis 194: 11–19. 16741877 10.1086/505147

[5] Adamson CS, Freed EO (2010) Novel approaches to inhibiting HIV-1 replication. Antiviral Res 85: 119–141. 19782103 10.1016/j.antiviral.2009.09.009 PMC2815006

[6] Adamson CS, Salzwedel K, Freed EO (2009) Virus maturation as a new HIV-1 therapeutic target. Expert Opin Ther Targets 13: 895–908. 19534569 10.1517/14728220903039714 PMC2737327

[7] Freed EO (1998) HIV-1 Gag proteins: diverse functions in the virus life cycle. Virology 251: 1–15. 9813197 10.1006/viro.1998.9398

[8] Ganser-Pornillos BK, Yeager M, Sundquist WI (2008) The structural biology of HIV assembly. Curr Opin Struct Biol 18: 203–217. 18406133 10.1016/j.sbi.2008.02.001 PMC2819415

[9] Erickson-Viitanen S, Manfredi J, Viitanen P, Tribe DE, Tritch R, et al (1989) Cleavage of HIV-1 gag polyprotein synthesized in vitro: sequential cleavage by the viral protease. AIDS Res Hum Retroviruses 5: 577–591. 2692658 10.1089/aid.1989.5.577

[10] Lee SK, Potempa M, Kolli M, Ozen A, Schiffer CA, et al (2012) Context surrounding processing sites is crucial in determining cleavage rate of a subset of processing sites in HIV-1 Gag and Gag-Pro-Pol polyprotein precursors by viral protease. J Biol Chem 287: 13279–13290. 22334652 10.1074/jbc.M112.339374 PMC3339993

[11] Tritch RJ, Cheng YE, Yin FH, Erickson-Viitanen S (1991) Mutagenesis of protease cleavage sites in the human immunodeficiency virus type 1 gag polyprotein. J Virol 65: 922–930. 1987379 10.1128/jvi.65.2.922-930.1991 PMC239833

[12] Ganser BK, Li S, Klishko VY, Finch JT, Sundquist WI (1999) Assembly and analysis of conical models for the HIV-1 core. Science 283: 80–83. 9872746 10.1126/science.283.5398.80

[13] Li S, Hill CP, Sundquist WI, Finch JT (2000) Image reconstructions of helical assemblies of the HIV-1 CA protein. Nature 407: 409–413. 11014200 10.1038/35030177

[14] Craven RC, Leure-duPree AE, Weldon RA Jr, Wills JW (1995) Genetic analysis of the major homology region of the Rous sarcoma virus Gag protein. J Virol 69: 4213–4227. 7769681 10.1128/jvi.69.7.4213-4227.1995 PMC189159

[15] Dorfman T, Bukovsky A, Ohagen A, Hoglund S, Gottlinger HG (1994) Functional domains of the capsid protein of human immunodeficiency virus type 1. J Virol 68: 8180–8187. 7966609 10.1128/jvi.68.12.8180-8187.1994 PMC237283

[16] McDermott J, Farrell L, Ross R, Barklis E (1996) Structural analysis of human immunodeficiency virus type 1 Gag protein interactions, using cysteine-specific reagents. J Virol 70: 5106–5114. 8764018 10.1128/jvi.70.8.5106-5114.1996 PMC190465

[17] Gamble TR, Yoo S, Vajdos FF, von Schwedler UK, Worthylake DK, et al (1997) Structure of the carboxyl-terminal dimerization domain of the HIV-1 capsid protein. Science 278: 849–853. 9346481 10.1126/science.278.5339.849

[18] Bowzard JB, Wills JW, Craven RC (2001) Second-site suppressors of Rous sarcoma virus Ca mutations: evidence for interdomain interactions. J Virol 75: 6850–6856. 11435564 10.1128/JVI.75.15.6850-6856.2001 PMC114412

[19] Cairns TM, Craven RC (2001) Viral DNA synthesis defects in assembly-competent Rous sarcoma virus CA mutants. J Virol 75: 242–250. 11119594 10.1128/JVI.75.1.242-250.2001 PMC113918

[20] Chang YF, Wang SM, Huang KJ, Wang CT (2007) Mutations in capsid major homology region affect assembly and membrane affinity of HIV-1 Gag. J Mol Biol 370: 585–597. 17532005 10.1016/j.jmb.2007.05.020

[21] Ebbets-Reed D, Scarlata S, Carter CA (1996) The major homology region of the HIV-1 gag precursor influences membrane affinity. Biochemistry 35: 14268–14275. 8916912 10.1021/bi9606399

[22] Mammano F, Ohagen A, Hoglund S, Gottlinger HG (1994) Role of the major homology region of human immunodeficiency virus type 1 in virion morphogenesis. J Virol 68: 4927–4936. 8035491 10.1128/jvi.68.8.4927-4936.1994 PMC236433

[23] Purdy JG, Flanagan JM, Ropson IJ, Rennoll-Bankert KE, Craven RC (2008) Critical role of conserved hydrophobic residues within the major homology region in mature retroviral capsid assembly. J Virol 82: 5951–5961. 18400856 10.1128/JVI.00214-08 PMC2395126

[24] Strambio-de-Castillia C, Hunter E (1992) Mutational analysis of the major homology region of Mason-Pfizer monkey virus by use of saturation mutagenesis. J Virol 66: 7021–7032. 1279197 10.1128/jvi.66.12.7021-7032.1992 PMC240357

[25] von Schwedler UK, Stray KM, Garrus JE, Sundquist WI (2003) Functional surfaces of the human immunodeficiency virus type 1 capsid protein. J Virol 77: 5439–5450. 12692245 10.1128/JVI.77.9.5439-5450.2003 PMC153941

[26] Willems L, Kerkhofs P, Attenelle L, Burny A, Portetelle D, et al (1997) The major homology region of bovine leukaemia virus p24gag is required for virus infectivity in vivo. J Gen Virol 78: 637–640. 9049415 10.1099/0022-1317-78-3-637

[27] Morellet N, Druillennec S, Lenoir C, Bouaziz S, Roques BP (2005) Helical structure determined by NMR of the HIV-1 (345–392)Gag sequence, surrounding p2: implications for particle assembly and RNA packaging. Protein Sci 14: 375–386. 15659370 10.1110/ps.041087605 PMC2253411

[28] Wright ER, Schooler JB, Ding HJ, Kieffer C, Fillmore C, et al (2007) Electron cryotomography of immature HIV-1 virions reveals the structure of the CA and SP1 Gag shells. EMBO J 26: 2218–2226. 17396149 10.1038/sj.emboj.7601664 PMC1852790

[29] Accola MA, Hoglund S, Gottlinger HG (1998) A putative alpha-helical structure which overlaps the capsid-p2 boundary in the human immunodeficiency virus type 1 Gag precursor is crucial for viral particle assembly. J Virol 72: 2072–2078. 9499062 10.1128/jvi.72.3.2072-2078.1998 PMC109501

[30] Datta SA, Temeselew LG, Crist RM, Soheilian F, Kamata A, et al (2011) On the role of the SP1 domain in HIV-1 particle assembly: a molecular switch? J Virol 85: 4111–4121. 21325421 10.1128/JVI.00006-11 PMC3126284

[31] Guo X, Roldan A, Hu J, Wainberg MA, Liang C (2005) Mutation of the SP1 sequence impairs both multimerization and membrane-binding activities of human immunodeficiency virus type 1 Gag. J Virol 79: 1803–1812. 15650204 10.1128/JVI.79.3.1803-1812.2005 PMC544129

[32] Krausslich HG, Facke M, Heuser AM, Konvalinka J, Zentgraf H (1995) The spacer peptide between human immunodeficiency virus capsid and nucleocapsid proteins is essential for ordered assembly and viral infectivity. J Virol 69: 3407–3419. 7745687 10.1128/jvi.69.6.3407-3419.1995 PMC189053

[33] Li F, Zoumplis D, Matallana C, Kilgore NR, Reddick M, et al (2006) Determinants of activity of the HIV-1 maturation inhibitor PA-457. Virology 356: 217–224. 16930665 10.1016/j.virol.2006.07.023

[34] Liang C, Hu J, Russell RS, Roldan A, Kleiman L, et al (2002) Characterization of a putative alpha-helix across the capsid-SP1 boundary that is critical for the multimerization of human immunodeficiency virus type 1 Gag. J Virol 76: 11729–11737. 12388733 10.1128/JVI.76.22.11729-11737.2002 PMC136778

[35] Fujioka T, Kashiwada Y, Kilkuskie RE, Cosentino LM, Ballas LM, et al (1994) Anti-AIDS agents, 11. Betulinic acid and platanic acid as anti-HIV principles from Syzigium claviflorum, and the anti-HIV activity of structurally related triterpenoids. J Nat Prod 57: 243–247. 8176401 10.1021/np50104a008

[36] Kanamoto T, Kashiwada Y, Kanbara K, Gotoh K, Yoshimori M, et al (2001) Anti-human immunodeficiency virus activity of YK-FH312 (a betulinic acid derivative), a novel compound blocking viral maturation. Antimicrob Agents Chemother 45: 1225–1230. 11257038 10.1128/AAC.45.4.1225-1230.2001 PMC90447

[37] Kashiwada Y, Hashimoto F, Cosentino LM, Chen CH, Garrett PE, et al (1996) Betulinic acid and dihydrobetulinic acid derivatives as potent anti-HIV agents. J Med Chem 39: 1016–1017. 8676334 10.1021/jm950922q

[38] Li F, Goila-Gaur R, Salzwedel K, Kilgore NR, Reddick M, et al (2003) PA-457: a potent HIV inhibitor that disrupts core condensation by targeting a late step in Gag processing. Proc Natl Acad Sci U S A 100: 13555–13560. 14573704 10.1073/pnas.2234683100 PMC263852

[39] Zhou J, Yuan X, Dismuke D, Forshey BM, Lundquist C, et al (2004) Small-molecule inhibition of human immunodeficiency virus type 1 replication by specific targeting of the final step of virion maturation. J Virol 78: 922–929. 14694123 10.1128/JVI.78.2.922-929.2004 PMC368845

[40] Adamson CS, Ablan SD, Boeras I, Goila-Gaur R, Soheilian F, et al (2006) In vitro resistance to the human immunodeficiency virus type 1 maturation inhibitor PA-457 (Bevirimat). J Virol 80: 10957–10971. 16956950 10.1128/JVI.01369-06 PMC1642185

[41] Adamson CS, Waki K, Ablan SD, Salzwedel K, Freed EO (2009) Impact of human immunodeficiency virus type 1 resistance to protease inhibitors on evolution of resistance to the maturation inhibitor bevirimat (PA-457). J Virol 83: 4884–4894. 19279107 10.1128/JVI.02659-08 PMC2682084

[42] Zhou J, Huang L, Hachey DL, Chen CH, Aiken C (2005) Inhibition of HIV-1 maturation via drug association with the viral Gag protein in immature HIV-1 particles. J Biol Chem 280: 42149–42155. 16251182 10.1074/jbc.M508951200

[43] Zhou J, Chen CH, Aiken C (2006) Human immunodeficiency virus type 1 resistance to the small molecule maturation inhibitor 3-O-(3′,3′-dimethylsuccinyl)-betulinic acid is conferred by a variety of single amino acid substitutions at the CA-SP1 cleavage site in Gag. J Virol 80: 12095–12101. 17035324 10.1128/JVI.01626-06 PMC1676313

[44] Sakalian M, McMurtrey CP, Deeg FJ, Maloy CW, Li F, et al (2006) 3-O-(3′,3′-dimethysuccinyl) betulinic acid inhibits maturation of the human immunodeficiency virus type 1 Gag precursor assembled in vitro. J Virol 80: 5716–5722. 16731910 10.1128/JVI.02743-05 PMC1472563

[45] Nguyen AT, Feasley CL, Jackson KW, Nitz TJ, Salzwedel K, et al (2011) The prototype HIV-1 maturation inhibitor, bevirimat, binds to the CA-SP1 cleavage site in immature Gag particles. Retrovirology 8: 101. 22151792 10.1186/1742-4690-8-101 PMC3267693

[46] Martin DE, Salzwedel K, Allaway GP (2008) Bevirimat: a novel maturation inhibitor for the treatment of HIV-1 infection. Antivir Chem Chemother 19: 107–113. 19024627 10.1177/095632020801900301

[47] Smith PF, Ogundele A, Forrest A, Wilton J, Salzwedel K, et al (2007) Phase I and II study of the safety, virologic effect, and pharmacokinetics/pharmacodynamics of single-dose 3-o-(3′,3′-dimethylsuccinyl)betulinic acid (bevirimat) against human immunodeficiency virus infection. Antimicrob Agents Chemother 51: 3574–3581. 17638699 10.1128/AAC.00152-07 PMC2043264

[48] McCallister S, Lalezari J, Richmond G, Thompson M, Harrigan R, et al.. (2008) HIV-1 Gag Polymorphisms Determine Treatment Response to Bevirimat (PA-457). In: Proceedings of the XVII International HIV Drug Resistance Workshop; 10–14 June 2008; Sitges, Spain.

[49] Seclen E, Gonzalez Mdel M, Corral A, de Mendoza C, Soriano V, et al (2010) High prevalence of natural polymorphisms in Gag (CA-SP1) associated with reduced response to Bevirimat, an HIV-1 maturation inhibitor. AIDS 24: 467–469. 19996935 10.1097/QAD.0b013e328335ce07

[50] Van Baelen K, Salzwedel K, Rondelez E, Van Eygen V, De Vos S, et al (2009) HIV-1 Susceptibility to the Maturation Inhibitor Bevirimat Is Modulated by Baseline Polymorphisms in Gag SP1. Antimicrob Agents Chemother 53: 2185–2188. 19223634 10.1128/AAC.01650-08 PMC2681549

[51] Adamson CS, Sakalian M, Salzwedel K, Freed EO (2010) Polymorphisms in Gag spacer peptide 1 confer varying levels of resistance to the HIV-1 maturation inhibitor bevirimat. Retrovirology 7: 36. 20406463 10.1186/1742-4690-7-36 PMC2873507

[52] Lu W, Salzwedel K, Wang D, Chakravarty S, Freed EO, et al (2011) A single polymorphism in HIV-1 subtype C SP1 is sufficient to confer natural resistance to the maturation inhibitor bevirimat. Antimicrob Agents Chemother 55: 3324–3329. 21502630 10.1128/AAC.01435-10 PMC3122462

[53] Blair WS, Cao J, Fok-Seang J, Griffin P, Isaacson J, et al (2009) New small-molecule inhibitor class targeting human immunodeficiency virus type 1 virion maturation. Antimicrob Agents Chemother 53: 5080–5087. 19805571 10.1128/AAC.00759-09 PMC2786326

[54] Keller PW, Adamson CS, Heymann JB, Freed EO, Steven AC (2010) HIV-1 maturation inhibitor bevirimat stabilizes the immature Gag lattice. J Virol 85: 1420–1428. 21106735 10.1128/JVI.01926-10 PMC3028903

[55] Freed EO, Orenstein JM, Buckler-White AJ, Martin MA (1994) Single amino acid changes in the human immunodeficiency virus type 1 matrix protein block virus particle production. J Virol 68: 5311–5320. 8035531 10.1128/jvi.68.8.5311-5320.1994 PMC236481

[56] Ono A, Demirov D, Freed EO (2000) Relationship between human immunodeficiency virus type 1 Gag multimerization and membrane binding. J Virol 74: 5142–5150. 10799589 10.1128/jvi.74.11.5142-5150.2000 PMC110867

[57] Ono A, Freed EO (1999) Binding of human immunodeficiency virus type 1 Gag to membrane: role of the matrix amino terminus. J Virol 73: 4136–4144. 10196310 10.1128/jvi.73.5.4136-4144.1999 PMC104193

[58] Ono A, Orenstein JM, Freed EO (2000) Role of the Gag matrix domain in targeting human immunodeficiency virus type 1 assembly. J Virol 74: 2855–2866. 10684302 10.1128/jvi.74.6.2855-2866.2000 PMC111776

[59] Ono A, Waheed AA, Joshi A, Freed EO (2005) Association of human immunodeficiency virus type 1 gag with membrane does not require highly basic sequences in the nucleocapsid: use of a novel Gag multimerization assay. J Virol 79: 14131–14140. 16254348 10.1128/JVI.79.22.14131-14140.2005 PMC1280195

[60] Taylor GM, Ma L, Vogt VM, Post CB (2010) NMR relaxation studies of an RNA-binding segment of the rous sarcoma virus gag polyprotein in free and bound states: a model for autoinhibition of assembly. Biochemistry 49: 4006–4017. 20387899 10.1021/bi902196e PMC2891057

[61] Bharat TA, Davey NE, Ulbrich P, Riches JD, de Marco A, et al (2012) Structure of the immature retroviral capsid at 8 A resolution by cryo-electron microscopy. Nature 487: 385–389. 22722831 10.1038/nature11169

[62] Waheed AA, Ono A, Freed EO (2009) Methods for the study of HIV-1 assembly. In: HIV Protocols, second edition. p. 163–184. 10.1007/978-1-59745-170-3_12 PMC6941941 19020825

[63] Brissault B, Kichler A, Guis C, Leborgne C, Danos O, et al (2003) Synthesis of linear polyethylenimine derivatives for DNA transfection. Bioconjug Chem 14: 581–587. 12757382 10.1021/bc0200529

[64] Adachi A, Gendelman HE, Koenig S, Folks T, Willey R, et al (1986) Production of acquired immunodeficiency syndrome-associated retrovirus in human and nonhuman cells transfected with an infectious molecular clone. J Virol 59: 284–291. 3016298 10.1128/jvi.59.2.284-291.1986 PMC253077

[65] Freed EO, Martin MA (1994) Evidence for a functional interaction between the V1/V2 and C4 domains of human immunodeficiency virus type 1 envelope glycoprotein gp120. J Virol 68: 2503–2512. 8139032 10.1128/jvi.68.4.2503-2512.1994 PMC236728

[66] Pornillos O, Ganser-Pornillos BK, Kelly BN, Hua Y, Whitby FG, et al (2009) X-ray structures of the hexameric building block of the HIV capsid. Cell 137: 1282–1292. 19523676 10.1016/j.cell.2009.04.063 PMC2840706

