# Supplementary figures and images for: Structural and Functional Insights into the HIV-1 Maturation Inhibitor Binding Pocket
Source: PLoS Pathog. 2012 Nov 8;8(11):e1002997. doi: 10.1371/journal.ppat.1002997 (PMC3493477; doi:10.1371/journal.ppat.1002997)

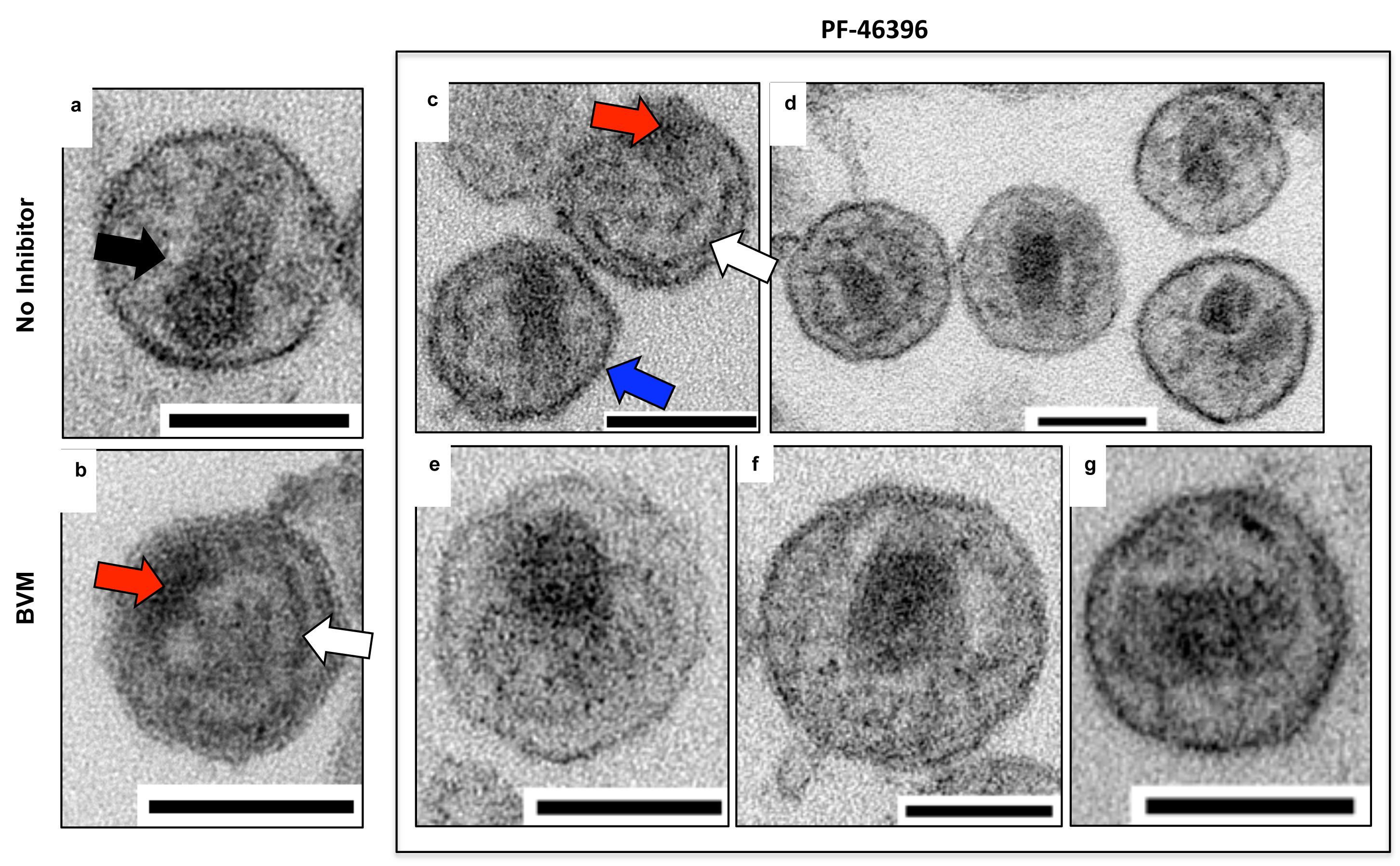

Supplement: Figure S1 — Morphology of virions produced from pNL4-3-transfected 293T cells in the absence of inhibitor (a), in the presence of 2 µM BVM (b), or 5 µM PF-46396 (c–g) by thin section transmission EM. Scale bar, 100 nm. Mature, conical cores are produced in the absence of inhibitor (black arrow). In the presence of maturation inhibitor, particles are observed to contain a crescent of electron density (white arrow) and a condensed aggregate of electron-dense material (red arrow). Particles produced from PF-46396 display greater morphological heterogeneity, including particles that contain both the electron-dense crescent and a condensed, conical core-like structure (blue arrow). (TIF) [file ppat.1002997.s001.tif]

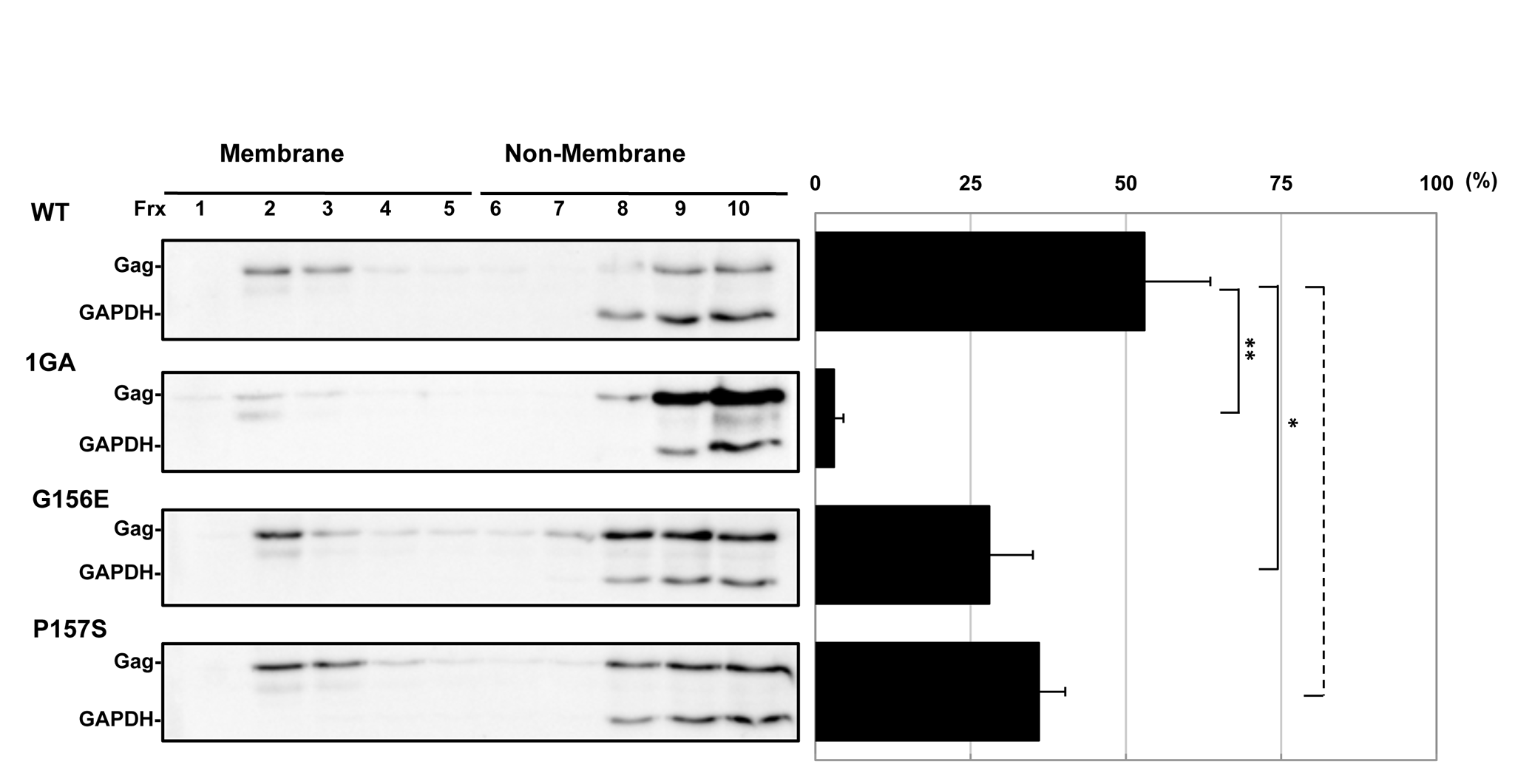

Supplement: Figure S2 — Membrane binding analysis for CA-G156E and P157S mutants. 293T cells were transfected with WT or mutant PR-defective (pNL4-3/PR−) molecular clones. Membrane flotation assays were performed as described in the Materials and Methods. Gag and GAPDH were detected by quantitative western blotting; bands were quantified by Imaging Lab software (Bio-Rad). WT and non-myristylated Gag (1GA) serve as positive and negative controls, respectively. P values: **, p<0.01; *, p<0.05. Dashed line indicates lack of statistical significance. N = 2–3. Frx = fraction. (TIF) [file ppat.1002997.s002.tif]

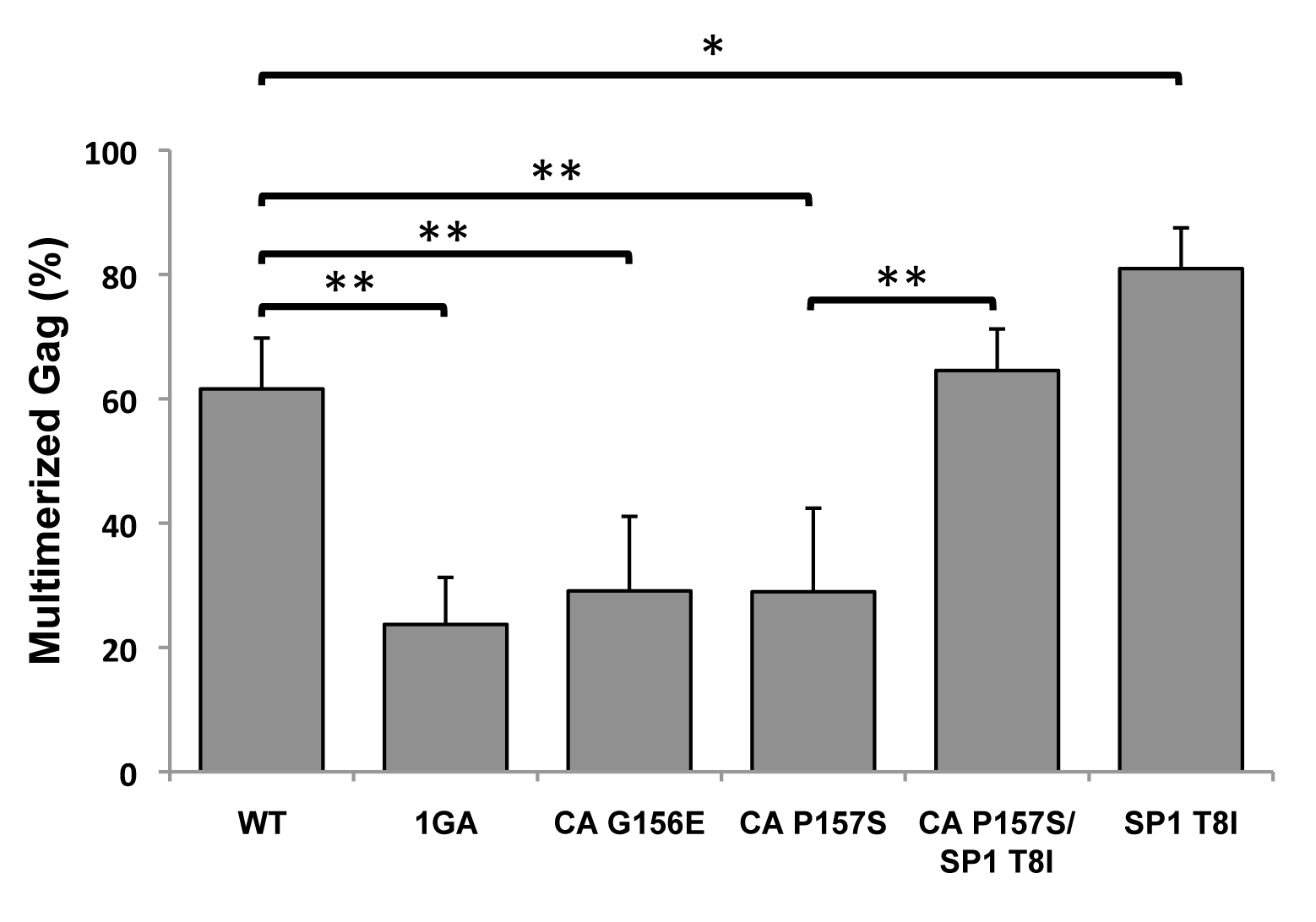

Supplement: Figure S3 — PF-46396-dependent MHR mutants are defective for Gag multimerization. Gag multimerization was evaluated in a cell-based assay described in the Materials and Methods. WT and the non-myristylated Gag mutant (1GA) served as positive and negative controls, respectively. P values: **, p<0.01; *, p<0.05. Note that the second-site compensatory mutation SP1-T8I rescues the multimerization defect imposed by the CA-P157S mutation. Error bars indicate SD; N = 3. (TIF) [file ppat.1002997.s003.tif]
